# Small‐molecule nanoprodrug with high drug loading and EGFR, PI3K/AKT dual‐inhibiting properties for bladder cancer treatment

**DOI:** 10.1002/EXP.20220141

**Published:** 2023-07-12

**Authors:** Guoyin Li, Zewen Song, Yi Ru, Jing Zhang, Lianxiang Luo, Wei Yang, Hao Wu, Haibao Jin, Xuanwen Bao, Di Wei, Zhao Yan, Haijing Qu, Zheng Zhu, Xiangdong Xue, Gang Zhou

**Affiliations:** ^1^ College of Life Science and Agronomy Zhoukou Normal University Zhoukou Henan China; ^2^ Department of Biochemistry and Molecular Biology State Key Laboratory of Cancer Biology The Fourth Military Medical University Xi'an Shaanxi China; ^3^ Department of Oncology Central South University Third Xiangya Hospital Changsha Hunan China; ^4^ Department of Pathology Xijing Hospital State Key Laboratory of Cancer Biology The Fourth Military Medical University Xi'an Shaanxi China; ^5^ The Marine Biomedical Research Institute Guangdong Medical University Zhanjiang Guangdong China; ^6^ Warshel Institute for Computational Biology School of Science and Engineering The Chinese University of Hong Kong Shenzhen China; ^7^ School of Basic Medical Sciences Xi'an Key Laboratory of Immune Related Diseases Xi'an Jiaotong University Xi'an Shaanxi China; ^8^ Shanghai Key Laboratory of Advanced Polymeric Materials School of Materials Science and Engineering East China University of Science and Technology Shanghai China; ^9^ Department of Medical Oncology The First Affiliated Hospital College of Medicine Zhejiang University Hangzhou Zhejiang China; ^10^ Graduate School Department of Biochemistry and Molecular Biology The Fourth Military Medical University Xi'an Shaanxi China; ^11^ School of Pharmacy Shanghai Jiao Tong University Xi'an Shanghai China; ^12^ Department of Medicine Harvard Medical School Boston Massachusetts USA; ^13^ National Translational Science Center for Molecular Medicine Department of Cell Biology State Key Laboratory of Cancer Biology Fourth Military Medical University Xi'an Shaanxi China

**Keywords:** bladder cancer, epidermal growth factor receptor (EGFR), hesperidin, high drug loading, triptolide

## Abstract

Bladder cancer (BCa) is one of the most common malignancies worldwide. Although multiple efforts have been made, the 5‐year survival rate of patients with BCa remains unchanged in recent years. Overexpression of the epidermal growth factor receptor (EGFR) is found in ≈74% of BCa tissue specimens; however, current EGFR‐based targeted therapies show little benefit for BCa patients, as the EGFR downstream pathways appear to be circumvented by other receptor tyrosine kinases (RTKs). In this study, two natural products are identified, namely triptolide (TPL) and hesperidin (HSP), that target and inhibit the EGFR and its downstream PI3K/AKT pathway in BCa. To synergistically combine triptolide and hesperidin, a succinic acid linker was employed to conjugate them and formed an amphiphilic TPL‐HSP EGFR‐targeting prodrug (THE), which further self‐assembled to generate nanoparticles (THE NPs). These NPs allowed the EGFR‐targeted delivery of the triptolide and hesperidin, and simultaneous inhibition of the EGFR and PI3K/AKT both in vitro and in vivo. This study provides a promising EGFR‐targeted delivery approach with the dual inhibition of the EGFR and PI3K/AKT, while also exhibiting a high drug loading and low toxicity. Our formulation may be a suitable option to deliver natural products for BCa treatment by EGFR‐targeted therapy.

## Graphical abstract

Overexpression of the EGFR is found in up to 74% of bladder cancer (BCa) tissue. Two natural products that were found, triptolide (TPL) and hesperidin (HSP), could inhibit EGFR pathways. They are linked together into a prodrug that can form nanoparticles in a water environment, which not only remarkably improved their bioavailability and therapeutic but also reduced their systemic toxicity.

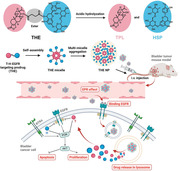

## 1 INTRODUCTION

Bladder cancer (BCa) is the 10th most commonly diagnosed cancer worldwide, with an estimated 573,000 new cases and 213,000 deaths in 2020.^[^ ^1^ ^]^ In the United States, the average 5‐year survival rate for patients with BCa is as high as 77.1%, but drops to 4.6% for those suffering from metastasis.^[^ ^2^ ^]^ Clinical treatments for BCa include surgery, chemotherapy, radiotherapy, targeted therapy, immunotherapy, and other traditional treatment approaches.^[^ ^3^ ^]^ When BCa becomes advanced, a cisplatin‐based combination chemotherapy regimen is recommended as the initial treatment; however, 50% of patients with advanced bladder cancer are not sufficiently fit to receive cisplatin‐based treatments because of its high toxicity. Therefore, identifying novel therapeutic options for patients with BCa is urgently required. In this context, the comprehensive genomic profiling of BCa has revealed a number of potential molecular targets, including the fibroblast growth factor receptor (FGFR) and the epidermal growth factor receptor (ErbB). Notably, expression of the epidermal growth factor receptor (EGFR, also known as ErbB1) is upregulated in approximately 74% of bladder carcinoma tissues compared to that in normal urothelial tissues.^[^ ^4^ ^]^ However, several clinical trials have shown that inhibiting of the EGFR has no benefit in BCa patients.^[^ ^5^ ^]^ These results indicate that inhibition of the EGFR in BCa may not be sufficient, or that the EGFR binds to other receptor tyrosine kinases (RTKs) to form heterodimers. Consequently, downstream pathways, including the PI3K/AKT and MAPK pathways, are activated to circumvent EGFR inhibition.^[^ ^6^ ^]^ Indeed, the drug lapatinib, which targets both the EGFR and ErbB2, was found to exhibit improved patient outcomes in the treatment of BCa, with a response rate of approximately 56%. However, in addition to such inhibition strategies, it is necessary to also focus on downstream pathways to allow the identification and development of novel EGFR‐targeting strategies for patients with BCa.

Natural products are known to exhibit various biological functions. For example, triptolide (TPL), which was first isolated from a perennial vine‐like Chinese medicinal herb (Tripterygium wilfordii Hook F (TwHF) or Thunder God Vine) in 1972,^[^ ^7^ ^]^ has been demonstrated to possess a wide range of pharmacological effects, including anti‐inflammatory and antitumor effects.^[^ ^8^ ^]^ More specifically, TPL plays an antitumor role by arresting cell cycle progression, triggering autophagy, and inducing apoptosis.^[^ ^9^ ^]^ Previous studies have shown that TPL exhibits antitumor activity by inhibiting EGFR pathways in multiple cancer types; however, the ability of TPL to inhibit the EGFR in BCa is yet to be examined. As another example natural product, hesperidin (HSP) is a flavonoid glycoside found abundantly in citrus fruits, and it has been reported to exhibit both antioxidant and anti‐inflammatory activities.^[^ ^10^ ^]^ Mounting evidence indicates that HSP can suppress tumor proliferation by inducing apoptosis and cell cycle arrest, while also inhibiting tumor cell migration and angiogenesis.^[^ ^11^ ^]^ Although HSP has been shown to potentiate antitumor effects by targeting the EGFR pathway in breast cancer, its role in BCa remains uninvestigated.^[^ ^12^ ^]^ Moreover, in addition to their inhibitory effects against the EGFR, TPL^[^ ^13^ ^]^ and HSP^[^ ^14^ ^]^ can also inhibit the downstream PI3K/AKT pathway of multiple RTKs. However, the applications of TPL and HSP in clinical use are limited by their high in vivo toxicities, poor aqueous solubilities, and poor bioavailabilities.

**FIGURE 1:**
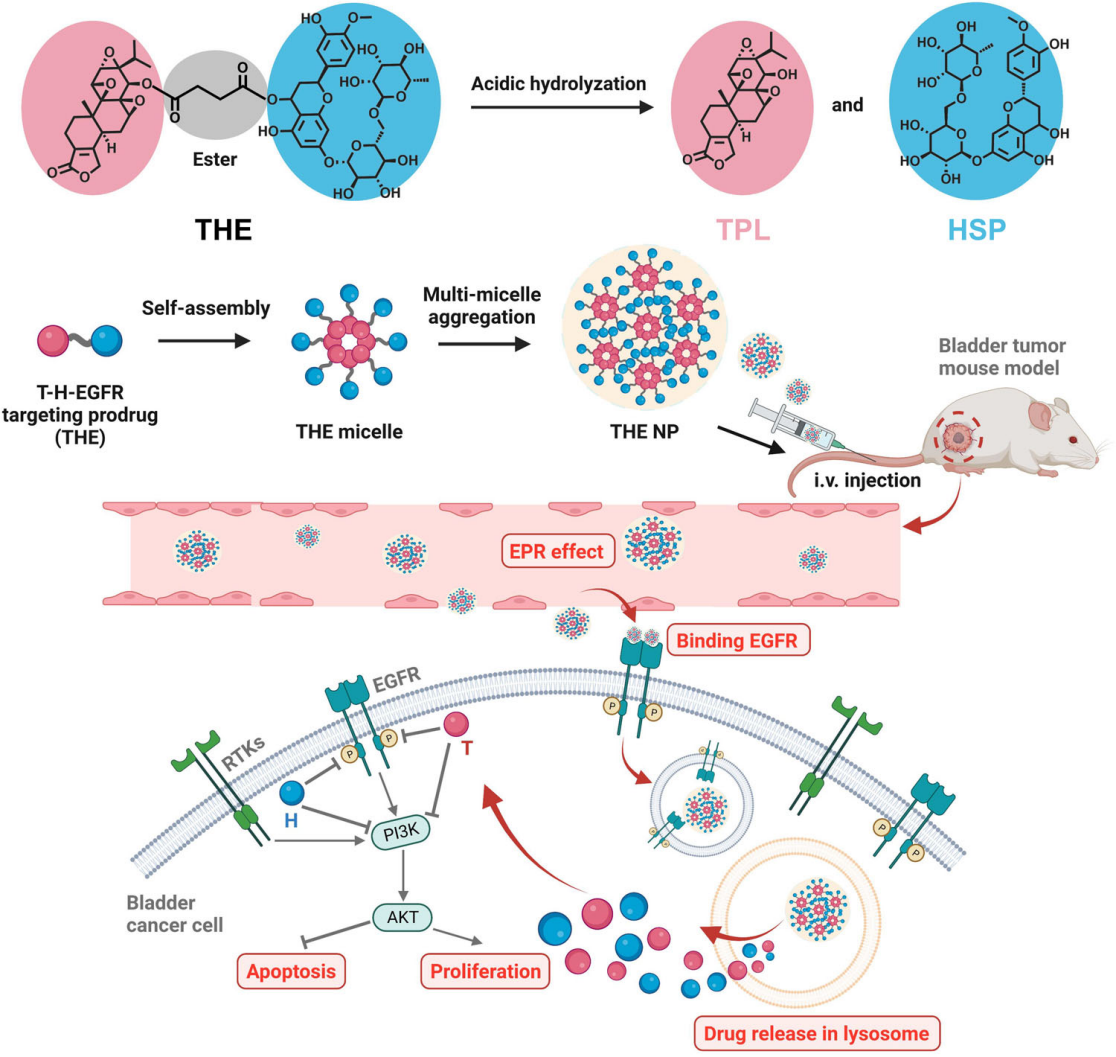
Schematic illustration of the synthesis, self‐assembly, and anti‐tumor mechanism of the THE NPs. The amphiphilic THE was synthesized and assembled into THE micelle, and further aggregated into large nanoparticle (THE NP) via multi‐micelle aggregation. THE NPs were i.v. administrated into bladder tumor‐bearing mice. THE NPs preferentially accumulated at the tumor site by taking advantage of EPR effect, then entered the tumor cells. The ester bond in THE prodrug can be readily cleaved by the acidic pH in the lysosome, making the THE NPs release the precursor drugs (TPL and HSP). TPL and HSP can both inhibit the EGFR and PI3K/AKT, trigger the consequent bioactivity, and therefore extensively suppress tumor growth.

Due to the beneficial properties of natural products, the application of natural product‐based nanoformulations in cancer treatment is an emerging field that is expected to address the issues outlined above. Nanoparticle‐based drug formulations show high versability which can extensively improve the druggability ^[^ ^15^ ^]^ and broaden the therapeutic modalities^[^ ^16^ ^]^ of small molecule drugs. By modifying the structures of natural products and controlling their sizes, the limitations related to water solubility, stability, and bioavailability can be effectively overcome.^[^ ^17^ ^]^ We hypothesized that the antioxidant effect and antitumor function of HSP could ameliorate the hepatorenal toxicity of TPL and enhance its antitumor effect, and so it was considered that a novel complex, integrating both HSP and TPL, could become a new and effective therapeutic for BCa treatment. Thus, using network pharmacological and molecular modeling to analyze the TPL and HSP co‐targeting genes, we initially demonstrate that the EGFR is a key gene in BCa. Surface plasmon resonance (SPR) experiments are employed to determine the affinities of HSP and TPL toward the EGFR, and subsequently, HSP and TPL are co‐assembled into single nanoparticles (NPs) for application in BCa therapy. As shown in Figure 1, HSP and TPL are conjugated via ester bonds to form an amphiphilic dual‐drug conjugate (i.e., the TPL‐HSP EGFR‐targeting prodrug, THE). The ester bond can be readily hydrolyzed at an acidic pH to yield the HSP and TPL, which should allow the drug precursors to exert their original anticancer effects. In addition, we propose that the amphiphilic THE conjugates will self‐assemble into tiny micelles prior to further aggregation to yield THE NPs via multi‐micelle aggregation that is likely to happen in the nanoassembly formed by small molecules.^[^ ^18^ ^]^ The affinity of the NPs toward the EGFR is determined in vitro using BCa 5637 cells, and after intravenous administration to bladder tumor‐bearing mice, the degree of NPs accumulation at the tumor site via the enhanced permeability and retention (EPR) effect of the solid tumor is evaluated. It is expected that once THE NPs arrive at the tumor site, they will be ingested by tumor cells and transported to the lysosome. The acidic pH of the lysosomes will then simultaneously release both drugs with the aim of inhibiting both the EGFR and PI3K/AKT. Finally, the ability of the prepared THE NPs to inhibit proliferation and induce apoptosis in BCa treatment is determined to evaluate the potential of this dual inhibition strategy for BCa‐targeted therapy.

## 2 MATERIALS AND METHODS

### 2.1 Materials and characterization

Triptolide was purchased from various commercial sources, including Tansoole and Meilunbio. All general chemicals were purchased from Adamas‐beta or Energy Chemical in the highest purity available and were used without further purification. The cell cycle and apoptosis detection kit, the total cell lysates (C1062L, Beyotime), the BCA protein concentration assay Kit, and the TUNEL cell apoptosis detection kit were purchased from Beyotime Biotechnology. The synthesized compounds were analyzed using a Bruker 400 MHz nuclear magnetic resonance (NMR) spectrometer. High‐resolution mass spectra (HRMS) were recorded using a ZAB‐HS spectrometer with electrospray ionization (ESI). High‐performance liquid chromatography (HPLC) analysis was performed using a Hanbo Sci&Tech NS4201 system equipped with an NU3000 serial UV/Vis detector and two NP7000 serial pumps. The binding affinity was investigated using a Biacore T200 SPR instrument (Cytiva, Sweden). The degrees of apoptosis and cell cycle progression were detected using a NovoCyte3130 flow cytometer (ACEA). Imaging was performed using confocal laser‐scanning microscopy (Nikon A1R).

### 2.2 Network pharmacology

The potential targets of HSP and TPL were acquired using PharmMapper,^[^ ^19^ ^]^ wherein the targets with *z*‐scores > 0 were reserved. GeneCards (https://www.genecards.org/)^[^ ^20^ ^]^ was used to screen the pathological targets of BCa. A Venn diagram was employed to identify the common targets of HSP and TPL for the treatment of BCa. Information related to the protein–protein interactions (PPIs) associated with the common targets was collected from the String database, wherein the species was limited to Homo sapiens. CytoHubba, a plug‐in of the Cytoscape 3.7.2 software package, was used to identify the hub genes based on their Degree, Betweenness, and Closeness values. Enrichment analysis of the Gene Ontology (GO) and Kyoto Encyclopedia of Genes and Genomes (KEGG) pathways was performed using DAVID 6.8.

### 2.3 Molecular docking

The structures of HSP, TPL, and THE were manually drawn using ChemDraw software and optimized in Chem3D using the FF94 force field prior to being saved in the mol2 database.^[^ ^21^ ^]^ SYBYL‐X docking software was employed, wherein the docking procedure was performed as previously described.^[^ ^17a^ ^]^

### 2.4 Cell culture and treatment

Human BCa 5637 cells were obtained from the Cell Bank of the Shanghai Institute for Biological Sciences (Chinese Academy of Sciences). The cells were cultured in PRMI1640 medium containing 10% FBS, and were maintained at 37°C in a humidified atmosphere containing 5% CO_2_. Cells were treated with HSP, TPL, and the THE NPs, which were prepared as described below.

### 2.5 General procedure for preparation of the nanoparticles

The nanoparticles were prepared using a single emulsion‐solvent evaporation method. More specifically, THE (30 mg) was dissolved in methanol (MeOH, 1.0 mL) then added dropwise to water (6 mL) and stirred for 0.5 h at room temperature. After this time, the MeOH solvent was removed with a rotary evaporator at 40°C to give the desired nanoparticles.

### 2.6 General procedure for preparation of the DiD‐loaded nanoparticles

THE (10 mg) was dissolved in MeOH (1.0 mL). After the addition of 1,1‐dioctadecyl‐3,3,3,3‐tetramethylindodicarbocyanine perchlorate (DiD, 0.5 mg), the resulting mixture was stirred at room temperature for 10 min then added dropwise to water (6 mL) and stirred for a further 0.5 h. After this time, the MeOH solvent was removed with a rotary evaporator at 40°C to give the DiD‐loaded nanoparticles.

### 2.7 CAC value of THE NPs was detected via pyrene radiometric method

1 µL of 0.1 mM pyrene solution (in acetone) was added to 999 µL of different concentrations of THE NPs solution and incubated at 37°C for 2 h. After incubation, the fluorescence of pyrene was evaluated by fluorescence spectrophotometer (excitation is 335 nm). The I3/I1 values were recorded for CAC evaluation.

### 2.8 The cumulative drug release assay

The release behavior of THE NPs was studied in PBS buffered solution (0.01 m) at three different pH values (pH 5.0 and 6.0, and physiological pH of 7.4). For this purpose, three sets of sample vials were taken, THE (10 mg) was placed and dissolved in methanol (MeOH, 1.0 mL) then added dropwise to water (6 mL) and stirred for 0.5 h. After this time, the MeOH solvent was removed with a rotary evaporator to give three groups THE NPs aqueous solution. The release studies were conducted in 4 mL of the corresponding buffer under shaking in an incubator at 37°C. At different time intervals, 20 µL of the sample was diluted with 1 mL of MeOH, filtered, and the TPL peak area was determined by HPLC. TPL (3.4 mg) was dissolved in 10 mL of MeOH, and 20 µl was taken and diluted with 1 mL of MeOH to measure the TPL peak area by HPLC, which was considered as 100% cumulative drug release control. HPLC conditions: acetonitrile/water (15:85) solution as mobile phase, 1 mL min^‐1^, retention time 36.8 min, 210 nm.

### 2.9 Apoptosis and cycle assays

A cell cycle and apoptosis detection kit (C1062L, Beyotime) and a NovoCyte3130 flow cytometer (ACEA) were used to analyze the cells. The apoptosis assay was performed by initially treating the cells with the drug compound or PBS for 24 h and then culturing in PRMI1640 medium containing 1% Fetal Bovine Serum (FBS) prior to fixation with pre‐cooled 70% ethanol for 4 h. The cells were subsequently stained with Annexin V‐FITC and propidium iodide for 15 min in the dark at 37°C and analyzed using a flow cytometer. The cell cycle assay was performed by initially culturing the cells in PRMI1640 medium containing 1% FBS for 12 h prior to treatment with the drug compounds. After culturing in PRMI1640 medium containing 10% FBS for 24 h, the cells were fixed with pre‐cooled 70% ethanol for 12 h and stained with propidium iodide for 15 min in the dark at 37°C. Finally, the cells were analyzed using a flow cytometer.

### 2.10 Western blotting

The total cell lysates were obtained using RIPA lysis buffer (P0013B, Beyotime). A BCA protein concentration assay kit (P0010S, Beyotime) was used to determine the protein concentration. The total protein specimen (30 µg) was resolved using sodium dodecyl sulfate–polyacrylamide gel electrophoresis and transferred onto a nitrocellulose membrane (GE Healthcare, Piscataway, NJ). The membrane was then probed with the following primary antibodies: EGFR (#4267, CST), Phospho‐EGFR (#3777, CST), AKT (#4685, CST), Phospho‐AKT (#4060, CST), PI3K (ab140307, Abcam), Phospho‐PI3K (AF3242, affinity), BCL2 (ab692, Abcam), BAX (ab53154, Abcam), SRC (#2019, CST), Phospho‐SRC (#12432, CST), and GAPDH (#2118, CST).

### 2.11 Immunohistochemistry and immunocytochemistry

The immunohistochemical and immunofluorescence analysis of the FFPE sections were performed as previously described.^[^ ^22^ ^]^ The Ki67 (ab16667, Abcam) antibody was used for immunostaining, while the BCL2, BAX, phospho‐EGFR, phospho‐PI3K, phospho‐AKT, and phospho‐SRC antibodies were used to achieve immunofluorescence. Imaging was performed using confocal laser scanning microscopy.

### 2.12 Lysosomal labeling

The 5637 cells were cultured as described above prior to incubation with the Lyso‐Tracker Red (C1046, Beyotime) staining working solution at 37°C for 30 min. After this time, Lyso‐Tracker Red removed, the cells were washed once with PBS, and observed by confocal laser scanning microscopy.

### 2.13 CCK‐8 assay

The CCK‐8 assay was performed as previously described.^[^ ^22^ ^]^ The 5637 cells were treated with different concentrations of TPL, HSP, and THE NPs for 48 h (concentrations = 0, 2, 10, 50, 250, 500, 1000, 2000, and 4000 nM). *n* = 4.

### 2.14 TUNEL assay

A TUNEL cell apoptosis detection kit was purchased from Beyotime Biotechnology (C1086). Paraffin tissue sections were dewaxed with xylene for 10 min (twice), then soaked in 90% ethanol for 2 min, 70% ethanol for 2 min, distilled water for 2 min. At room temperature, the tissues were treated with 20 µg·mL^−1^ of proteinase K without DNase for 20 min and washed three times with PBS. TUNEL solution (100 µL) was added to the sample and incubated at 37°C for 60 min in the dark. The samples were examined by confocal laser scanning microscopy.

### 2.15 Mouse models and treatment

The mice used in this study were purchased from the Model Animal Research Center of Nanjing University (China). All in vivo experiments were performed according to the protocols approved by the Laboratory Animal Center of Xi'an Jiaotong University and were in accordance with the policies of the National Ministry of Health (No. 2021–209). Six‐week‐old BALB/c female nude mice were used to establish xenograft BCa models. A total of 5637 cells (1 × 10^7^ per mouse) were injected subcutaneously into the mice, and the mice were randomly divided into five groups of five mice each. After 7 days (Day 0), the mice were treated with PBS, 0.5 mg kg^−1^ TPL, 0.85 mg kg^−1^ HSP, 0.5 mg kg^−1^ TPL and 0.85 mg kg^−1^ HSP, 1.5 mg kg^−1^ THE NPs, or PBS via the tail vein. Over days 1−15, the mice were subjected to repeated treatment at 2 days intervals. The tumor volume was measured every 48 h, and the weights of the mice were measured twice a week. Ten‐week‐old C57 mice were used to establish the acute poisoning model. The mice were treated with 5 mg kg^−1^ TPL, 8.5 mg kg^−1^ HSP, 5 mg kg^−1^ TPL and 8.5 mg kg^−1^ HSP, 15 mg kg^−1^ THE NPs, or PBS via the tail vein. After 24 h of drug treatment, the peripheral blood of the mice was sampled to carry out blood routine tests, and the serum was taken to determine the alanine aminotransferase (ALT), aspartate aminotransferase (AST), alkaline phosphatase (ALP), total bilirubin (TB), direct bilirubin (DB), creatinine (Cr), α‐hydroxybutyrate dehydrogenase (HBDH), phosphocreatine kinase (PK), γ‐glutamyl transferase (GTT), uric acid (UA), creatine kinase (CK), total protein (TP), lactate dehydrogenase (LDH), and albumin (AIB) contents. The livers and kidneys of the mice were used for HE staining to evaluate the biosafety of each drug compound/combination.

### 2.16 Statistical analysis

Data were analyzed using GraphPad prism software as follows: for experiments including apoptosis, cell cycle, mouse models, statistical significance was evaluated using the two‐tailed Student's *t*‐test, with *p* < 0.05 considered significant.

## 3 RESULTS AND DISCUSSION

### 3.1 EGFR targeting of HSP and TPL in BCa

**FIGURE 2:**
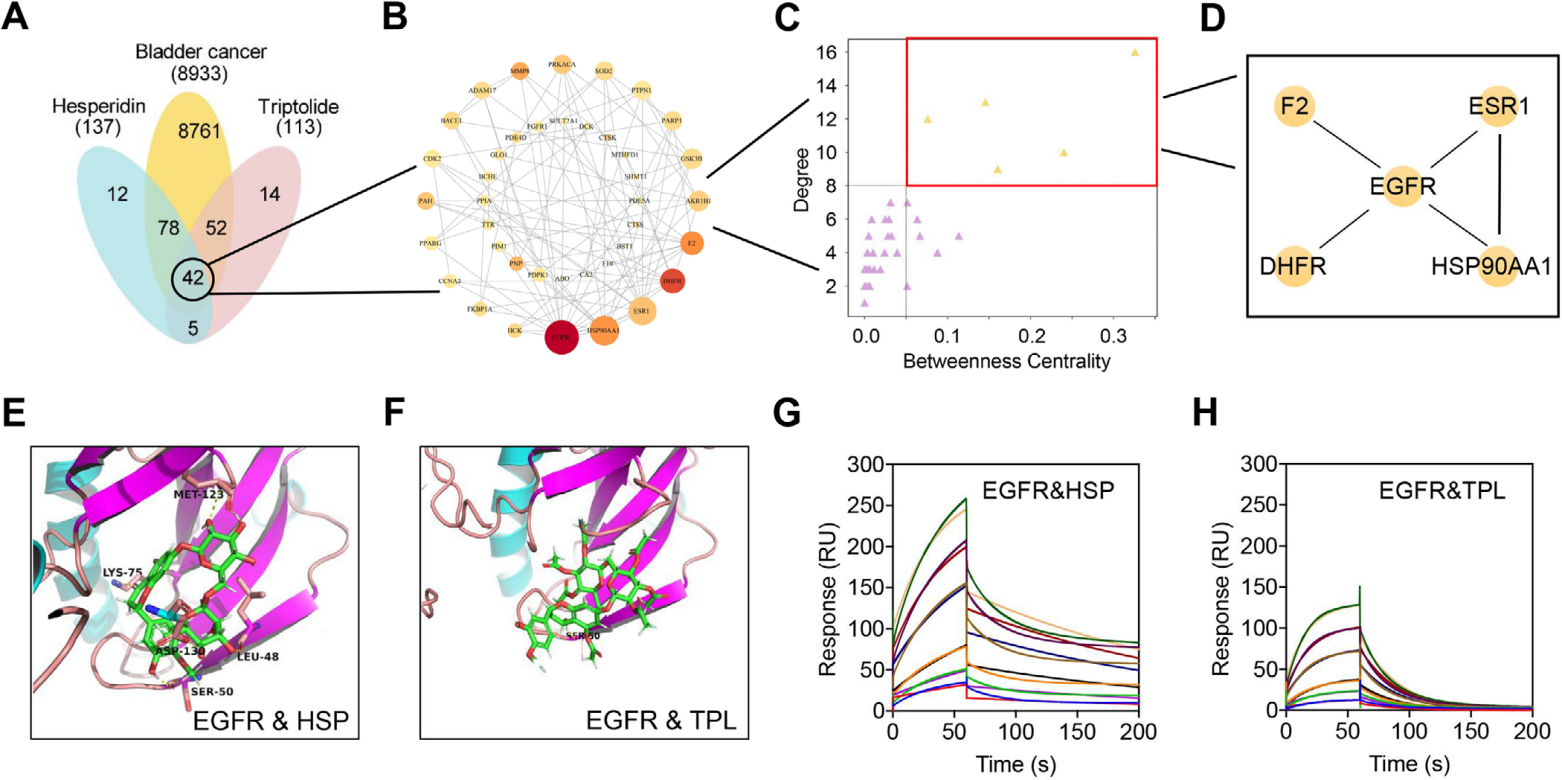
HSP and TPL can bind to EGFR in BCa. Network pharmacological analysis of HSP combined with TPL against BCa: (A) Venn diagram showed 42 common targets of hesperidin combined with triptolide treating BCa; (B) PPI network of common targets; (C,D) Hub genes in common targets. (E,F) The docked pose of the HSP and TPL in the EGFR ligand binding pocket. (G,H) The SPR experiment showed that HSP and TPL exhibited binding affinity to the truncated EGFR^25‐645.^

To search for common target genes of HSP and TPL in BCa, network pharmacological analysis was performed, and 42 common target genes were identified (Figure 2A). The PPI network of common targets contained 40 nodes (two targets were not related to this network) and 93 edges, with an average node degree of 4.43 (Figure 2B). CytoHubba was used to identify hub genes in the common targets, which were ranked based on their Degree, Betweenness, and Closeness (Figure 2C,D), wherein a darker color indicates a greater Degree, while a larger node size relates to an improved Betweenness. Thus, the EGFR, ESR1, HSP90AA1, DHFR, and F2 were identified as hub genes for the combined treatment of BCa with HSP and TPL. As mentioned above, the EGFR acts as a key driver of BCa and thus exhibits great potential as a therapeutic target for the treatment of BCa.^[^ ^23^ ^]^

To confirm whether HSP and TPL can bind to the intracellular domain of the EGFR, simulated molecular docking was employed to mimic their interactions with the EGFR. More specifically, HSP and TPL were docked into the ligand‐binding pocket of the EGFR, as shown in Figure 2E,F, and considerably good binding affinities were recorded (pIC_50_ = 7.09 and 7.15 towards the EGFR, respectively). To confirm that HSP and TPL directly target the EGFR in vitro, SPR experiments were carried out. It was found that HSP and TPL exhibited good binding affinities for the truncated EGFR (aa.25‐645, extracellular domain) with estimated KD constants of 0.921 and 3.483 mm, respectively (Figure 2G,H). The results of bioinformatics analysis, molecular docking and SPR were highly consistent, thereby suggesting that the EGFR is a potential target for the combined HSP‐TPL treatment of BCa.

### 3.2 Preparation and characterization of the THE NPs

**FIGURE 3:**
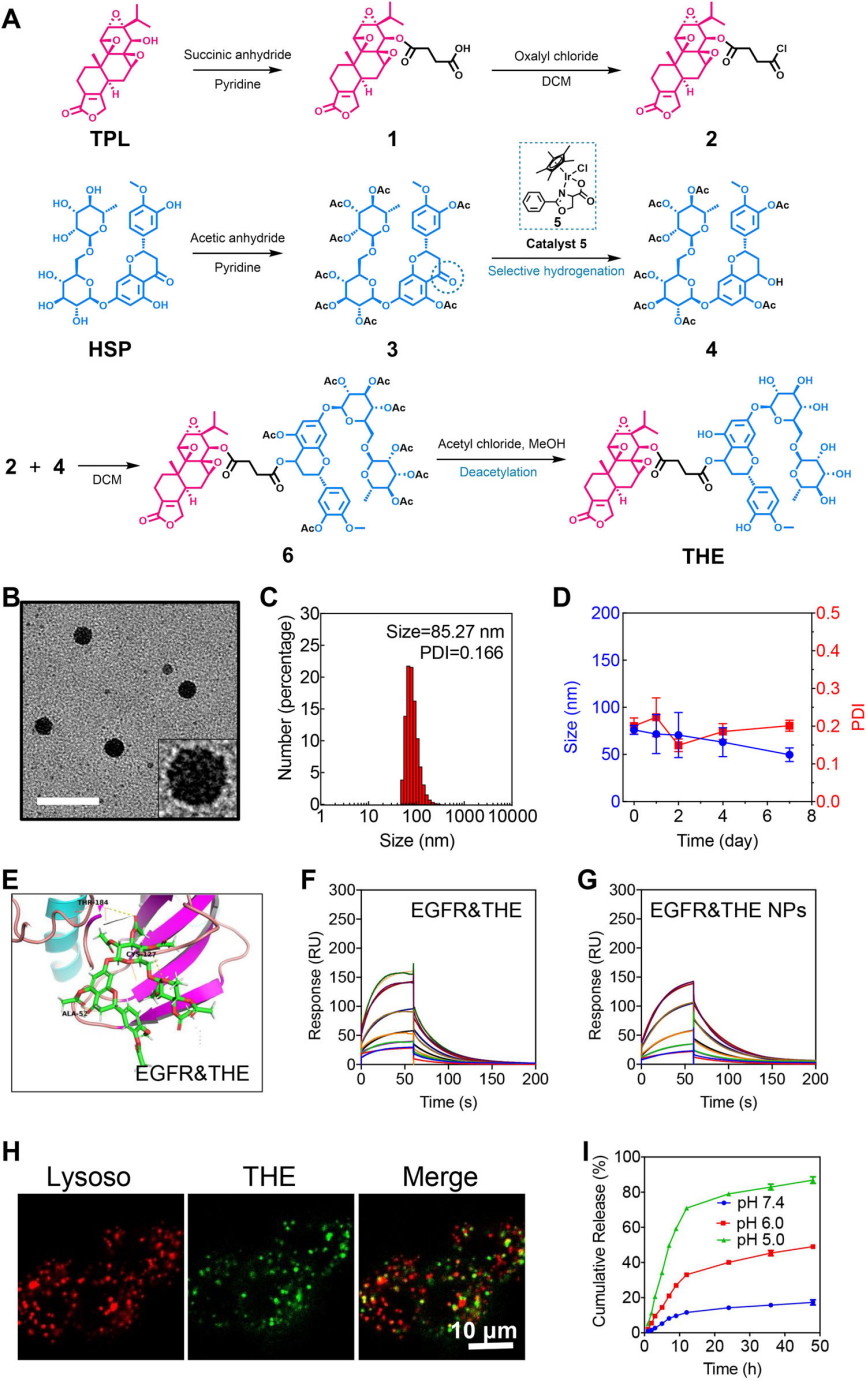
Synthesis and characterization of THE NPs / THE NPs are acidolysis into HSP and TPL in lysosomes. (A)The synthetic route of THE. (B) TEM image of THE NPs nanoparticles. The scale bar is 100 nm. (C) The size distribution of THE NPs monitored by DLS. (D) Serum stability of THE NPs based on size changes PDI variations. (E) The docked pose of THE in the EGFR ligand binding pocket. The SPR experiment showed that (F) THE and (G) THE NPs exhibited binding affinity to the truncated EGFR25‐645. (H) The colocalization of THE NPs with lysosomes was observed by CLSM. A hydrophobic dye (DiD) was encapsulated in THE NPs to make them traceable. The lysosomes were stained by LysoTracker. The scale bar is 50 µm. (I) The release rate of TPL increased with the decrease in pH.

As described above, TPL and HSP share a common target, namely the EGFR, and it is possible that the antioxidant HSP may suppress the oxidative toxicity of TPL to the kidney and liver. Such synergistic integration of TPL and HSP may therefore result in a superior anticancer efficacy and an improved biocompatibility. Thus, with this in mind, TPL and HSP were covalently conjugated into a prodrug (THE) and assembled into NPs with the aim of improving their in vivo performance. As outlined in Figure 3A, in the presence of a catalytic amount of 4‐dimethylaminopyridine (DMAP), TPL was reacted with succinic anhydride in pyridine to yield compound **1** (Figures S1,S2).^[^ ^24^ ^]^ In addition, HSP was reacted with an excess of acetic anhydride to synthesize the acetylated HSP (compound **3**, Figures S3,S4), and the carbonyl group of the acetylated HSP was selectively reduced to a hydroxyl group by transfer hydrogenation to afford compound **4** (Figures S5,S6) in the presence of catalyst **5** ^[^ ^25^ ^]^ (Figures S7,S8). It should be noted that compound **4** was not obtained using traditional reductants, such as NaBH_4_ or LiAlH_4_, or even using conventional palladium‐ or platinum‐based catalysts. Furthermore, compound **1** was converted to compound **2** using oxalyl chloride, and then reacted with compound **4** in dry dichloromethane to form compound **6**. After removal of the solvent without further purification, **6** was efficiently deacetylated using a previously reported method to obtain THE.^[^ ^26^ ^]^ The successful synthesis of THE was confirmed using HRMS and ^1^H and ^13^C NMR spectroscopy (Figures S9,S10). A molecular docking assay and SPR experiment were then employed to investigate whether the THE conjugate exhibited a high affinity for the EGFR. It was found that the amphiphilic THE conjugates readily self‐assembled into tiny micelles and further aggregated into large NPs via multi‐micelle aggregation. These NPs exhibited a spherical morphology, as observed by transmission electron microscopy (TEM) (Figure 3B); the inset of the TEM micrograph shows that the THE NPs harbored many small nanoparticles, which is highly consistent with the self‐assembly process outlined in Figure 1. Moreover, dynamic light scattering (DLS) experiments revealed that the THE NPs were uniform in size (polydispersity index, PDI = 0.166) with an average hydrodynamic diameter of 85 nm (Figure 3C). The harbored small micelles were measured as ≈7 nm in size based on the TME micrograph. The critical aggregation concentration (CAC) of THE NPs was measured by pyrene radiometric method, as shown in Figure S11, the CAC of THE NPs was 3.64 µm; the low CAC indicated that THE NPs still remain the nanostructure upon the blood dilution after the i.v. injection. The THE NPs also exhibited a good serum stability, as shown in Figure 3D, wherein both the particle size and PDI fluctuated within narrow ranges during a period of 1 week. These NPs not only addressed the poor water solubility of TPL, but they also exhibited an overwhelmingly higher drug loading (HSP 57.94%, TPL 34.05%, total loading = 91.99%) than the currently developed HSP‐^[^ ^27^ ^]^ and TPL‐based^[^ ^28^ ^]^ NPs.

### 3.3 Endocytosis and acidolysis of the THE NPs

Previous studies have shown that TPL and HSP can individually bind to the EGFR, and so the affinity between the prepared THE and the EGFR was subsequently investigated. Molecular docking studies showed that THE possessed a good binding affinity (−8.64 kcal mol^−1^) toward the EGFR with converted Kd of 2.29 nm (Figure 3E), as confirmed by SPR analysis (Figure 3F). The affinity of the THE NPs toward the EGFR was then examined using an SPR assay (Figure 3G), wherein it was demonstrated that the NPs could bind to the truncated EGFR^25−645^ with an estimated KD constant of 0.789 mm. It should be noted that this value indicates a weaker binding than those exhibited by the THE conjugate (Figure 3F) and the drug precursors (HSP and TPL) (Figure 2G,H). It was therefore considered that this effect was due to the larger size of the THE NPs compared to the conjugate and the drug precursors, which could lead to a larger penetration effect. Overall, these results demonstrate that the prepared THE NPs can successfully bind to the EGFR in BCa cells.

After binding to the EGFR, the THE NPs were supposed to be internalized by BCa cells. To verify the cell uptake, a hydrophobic dye (DiD) was encapsulated in the THE NPs to make them fluorescently traceable and the cell membrane was stained as well. As shown in Figure S12, the THE NPs were gradually internalized into cells (move away from the membrane) with time elapse, demonstrating that the THE NPs can be effectively uptaken into the cells. Generally, nanoparticles are transported into lysosomes after being internalized. Hence, the THE NPs were incubated with BCa cells, and their fluorescence was co‐localized with the lysosomes (indicated by LysoTracker, Figure 3H), demonstrating that the THE NPs were successfully ingested by cells and subsequently transported into the lysosomes. This fact reflected that the THE NPs may release the free drugs by taking advantage of the acidic lysosomal pH as the THE monomer was prepared by the formation of an ester bond between TPL and HSP. The drugs should escape from the lysosomes to affect the cells, therefore, we investigated the lysosome escaping process by incubate the THE NPs with BCa cells in different time interval. As shown in Figure S13, the THE NPs were first showed large colocalization area with lysosomes, then gradually detached from the lysosomes, demonstrating that the THE NPs can escape from the lysosomes.

The cumulative drug release was investigated by monitoring the acidolysis rate of the THE NPs at different pH values (5.0, 6.0, and 7.4) using HPLC. As shown in Figure 3I, the THE NPs remained stable in neutral pH, while the reduction in pH can extensively expedite the drug release. More specifically, at pH 6.0, approximately 50% of the TPL was released after 48 h of incubation, while at pH 5.0, approximately 75% of the TPL was released in the first 12 h, although this amount gradually increased over the following 36 h. Considering that the lysosome interior possesses an acidic pH of ≈5.0, these drug release results demonstrate that the THE NPs are able to load the drug conjugate at physiological pH and responsively release both HSP and TPL following endocytosis by the lysosomes. In contrast, after incubation for 48 h in PBS buffer, <20% of drug release was observed from the THE NPs, indicating their high stability under neutral pH conditions.

### 3.4 THE NPs inhibit proliferation and promote the apoptosis of BCa cells

**FIGURE 4:**
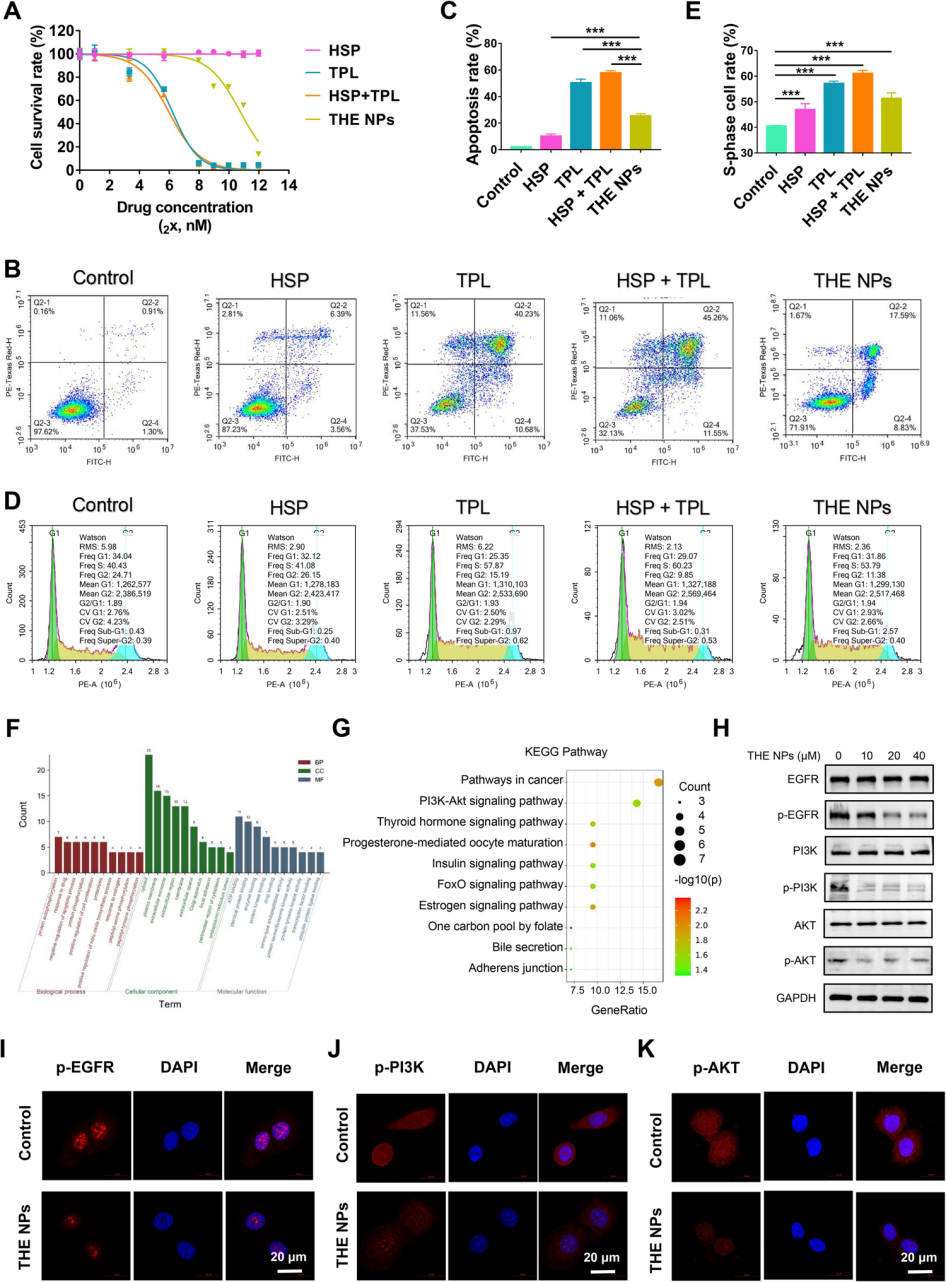
THE NPs inhibits BCa cell proliferation and promotes apoptosis by targeting EGFR. (A) The NPs can inhibit the activity of PI3K / Akt signaling pathway in BCa. The 5637 cells were treated with the indicated dose of PBS, HSP, TPL, HSP+TPL and THE NPs for 48 h. Cell survival rates were calculated and plotted. (B–E) The 5637 cells were treated with the indicated dose of PBS, HSP (10 µm), TPL (10 µm), HSP (10 µm) + TPL (10 µm) and THE NPs (10 µm) for 24 h, and were subject to flow cytometry assay for (B,C) apoptosis and (D,E) cell cycle. (F) GO and (G) KEGG enrichment analysis of the 42 common targets obtained by grid pharmacological analysis. (H) The 5637 cells were treated with indicated dose of THE NPs for 24 h and were subject to western blot analyses. (I–K) Immunofluorescence analysis of 5637 cells treated with THE NPs (10 µm) for 24 h. The scale bar is 20 µm. *p < 0.05; **p < 0.01; ***p < 0.001.

Previous studies have shown that HSP^[^ ^10b^ ^]^ and TPL^[^ ^8b^ ^]^ play antitumor roles in various cancers. Thus, to investigate the antitumor effects of the prepared THE NPs, in vitro experiments were carried out. Firstly, the CCK‐8 assay demonstrated that low‐dose TPL significantly inhibited the proliferation of 5637 cells, which was consistent with previous reports (Figure 4A).^[^ ^8^, ^29^ ^]^ In contrast to TPL, HSP showed mild cytotoxicity because due to the fact that the anticancer effect of HSP is mainly related to its antioxidant and anti‐inflammatory activities.^[^ ^10b^ ^]^ In addition, the inhibitory effect exhibited by HSP was almost undetectable due to the fact that the treatment dose in the CCK‐8 assay was far below its IC_50_ value. A physical mix of HSP and TPL exhibited similar antitumor effects to those of TPL alone, indicating that free mixture didn't improve the antitumor efficacy of TPL. Furthermore, the THE NPs showed reduced cytotoxicity compared to the free TPL since drug release was slowed down in the NP system. Subsequently, the effects of the NPs on apoptosis and the cell cycle were evaluated. For this purpose, 5637 cells were treated with PBS, HSP, TPL, HSP + TPL, and NPs and subjected to flow cytometry analysis. The results showed that the THE NPs significantly induced apoptosis and S‐phase arrest in the 5637 cells, and the observed activity was more potent than that of HSP but weaker than that of TPL (Figure 4B–E). It was therefore considered that the NPs trigger DNA damage and/or methionine restriction to induce S‐phase arrest in the 5637 cells.^[^ ^30^ ^]^ We also applied colony‐forming assay and 5‐ethynyl‐2′‐deoxyuridine (EdU) assay to evaluate the cell proliferation inhibition of THE NPs, respectively. Colony‐forming assay showed that THE NPs could efficiently inhibit 5637 cells forming colony and EdU test showed that THE NPs significantly inhibit proliferation of 5637 cells (Figure S14). These results indicate that the THE NPs inhibit proliferation and promote the apoptosis of BCa cells by targeting the EGFR, which is overexpressed in ≈74% of BCa cases,^[^^4b]^ thereby rendering it a potential target for clinical use.

### 3.5 Mechanism of THE NP‐induced apoptosis of the BCa cells

HSP and TPL were subjected to GO and KEGG analysis using DAVID6.8, and the top 10 GO enrichment results for the biological processes, cellular components, and molecular functions are presented in Figure 4F, including protein autophosphorylation, the negative regulation of apoptosis, and the positive regulation of cell proliferation. Ten KEGG pathways were retained after screening at a *p*‐value of <0.05. Interestingly, the PI3K/AKT signaling pathway was also co‐targeted by TPL and HSP (Figure 4G). Although the EGFR is widely expressed in BCa, it has been reported that targeting this receptor provides little benefit to patients with BCa. One possible reason for this is that the downstream PI3K/AKT pathway could be activated by other RTKs, such as FGFR. Numerous studies have shown that the PI3K/AKT pathway plays a crucial role in the proliferation and migration of BCa,^[^ ^31^ ^]^ and thus inhibition of the PI3K/AKT pathway could potentially inhibit BCa cell proliferation and promote apoptosis.^[^ ^32^ ^]^

To confirm these bioinformatic results, TPL and HSP were tested for EGFR inhibition in 5637 cells. It was found that TPL and HSP effectively inhibited phosphorylation of the EGFR, which is consistent with the results of previous studies (Figure S15).^[^ ^33^ ^]^ To investigate whether the cell killing effect would be weakened after knocking down EGFR or inhibiting EGFR activity, We applied siRNA in 5637 cells to knockdown the expression of EGFR, siEGFR efficacy was tested via Western blot. As shown in Figure S16, the killing effect of THE NPs on bladder cancer was weakened after knocking down EGFR, demonstrating that EGFR played a vital role in our nanoparticle to interact with BCa cells. Subsequently, the ability of the THE NPs to inhibit the activity of the EGFR/PI3K/AKT pathway was investigated by treating 5637 cells with the prepared NPs. As a result, the ICC assay demonstrated that the phosphorylation levels of the EGFR, PI3K, and AKT decreased significantly after NP treatment (Figure 4H), as supported by the observed protein levels (Figure 4I–K). These results suggested that the THE NPs could inhibit the activity of the EGFR/PI3K/AKT signaling pathway in 5637 cells. To investigate whether their signal pathways compensate for the EGFR activation of PI3K, We treated the 5637 cell line for 48 h in different concentrations and tested protein expression levels via Western blot afterwards. As shown in Figure S17, THE NPs can effectively inhibit the activity of SRC, thereby inhibiting its compensation for EGFR signal. The levels of apoptosis‐related proteins were also tested, and it was found that BCL2 was significantly downregulated, whereas BAX was significantly upregulated in the 5637 cells after THE NPs treatment (Figure S18). Therefore, it appeared that the THE NPs could simultaneously target the EGFR and PI3K/AKT to inhibit BCa growth and induce apoptosis.

### 3.6 In vivo antitumor effect and biocompatibility of the THE NPs

**FIGURE 5:**
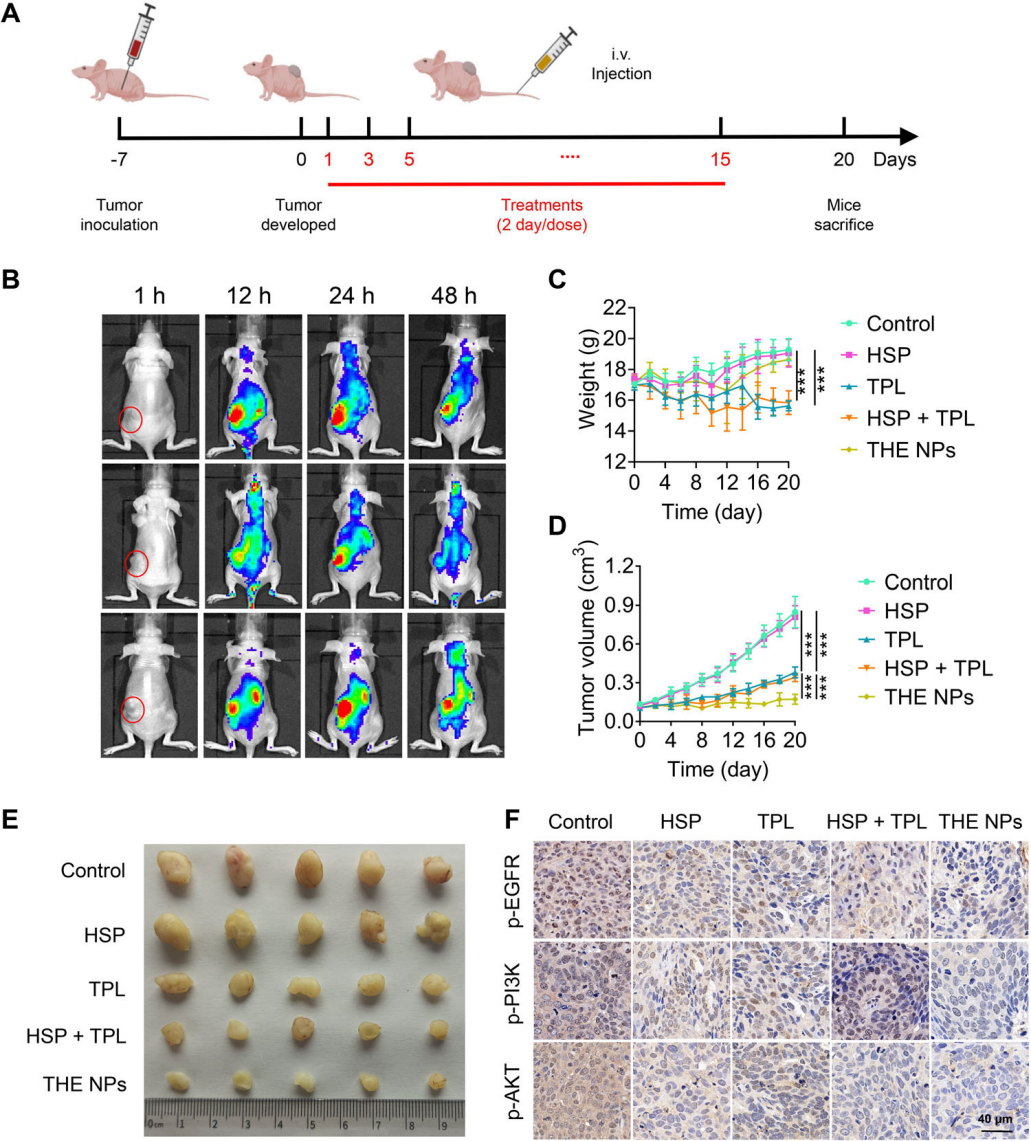
THE NPs can suppress BCa in vivo. (A) Schematic diagram of establishment and treatment of xenograft BCa models. (B) Distribution of THE NPs in different time periods after caudal vein injection indicated by DiD. (C,D) mice bearing subcutaneous BCa tumor after different treatments, body weight and tumor volume, *n* = 5. (E) Mice were sacrificed after 20 days of treatment and obtained BCa tissues. (F) Immunofluorescence of p‐EGFR, p‐PI3K, and p‐AKT analysis of cancer tissues from xenograft BCa models after 20 days of treatment with PBS, HSP, TPL, HSP + TPL, and THE NPs. The scale bar is 40 µm. *p < 0.05; **p < 0.01; ***p < 0.001.

Before we conducted the in vivo antitumor effect, the pharmacokinetics (PK) of THE NPs versus TPL were evaluated to see if the nanoformulation can improve the blood circulation of the hydrophobic drug. As shown in Figure S19, The AUC of THE NPs (14.69) was 8.96 times larger than free TPL (1.638), while the half circulation time of THE NPs (4.9) was 5.4 times longer than free TPL (0.9 h). The PK study supported that our THE NPs can remarkably improve the blood circulation of the hydrophobic TPL. To investigate the antitumor effects of the NPs in vivo, xenograft BCa mouse models were injected with PBS, HSP, TPL, HSP+TPL, and the NPs into the tail vein (Figure 5A). As shown in Figure 5B, the THE NPs preferentially accumulated at the tumor site after 12 h, indicating that the NPs could effectively enter the tumor tissue due to enhanced permeability and retention. The body weights of the mice were also monitored, and no significant differences were found among the control, HSP, and THE NPs groups, while the TPL and HSP + TPL groups were significantly decreased (Figure 5C). These results indicate that TPL exhibits substantial biological toxicity, while our developed nanoformulation (THE NP) can significantly improve the dose tolerance toward TPL. Upon monitoring the tumor growth status, the tumor volume in the THE NPs 0.173 ± 0.039 cm^3^ on day 20, which was significantly lower than the volumes of 0.847 ± 0.122 cm^3^, 0.81 ± 0.089 cm^3^, 0.381 ± 0.043 cm^3^ and 0.314 ± 0.032 cm^3^ found in the control, HSP, TPL and HSP + TPL groups, further demonstrating that the THE NPs strongly inhibited tumor proliferation (Figure 5D,E). Subsequently, the tumors were collected and the expression levels of the phosphorylated EGFR, PI3K and AKT were determined using immunohistochemistry (IHC). It was found that the phosphorylation level of the EGFR, PI3K and AKT in the THE NP‐treated group was significantly lower than that of the other groups, which was consistent with the results of the cytological experiments, again indicating that the THE NPs played an anticancer role by co‐targeting the EGFR and PI3K/AKT (Figures 4H–K and 5F). These results demonstrated that the THE NPs effectively inhibit and kill BCa cells in vivo.

**FIGURE 6:**
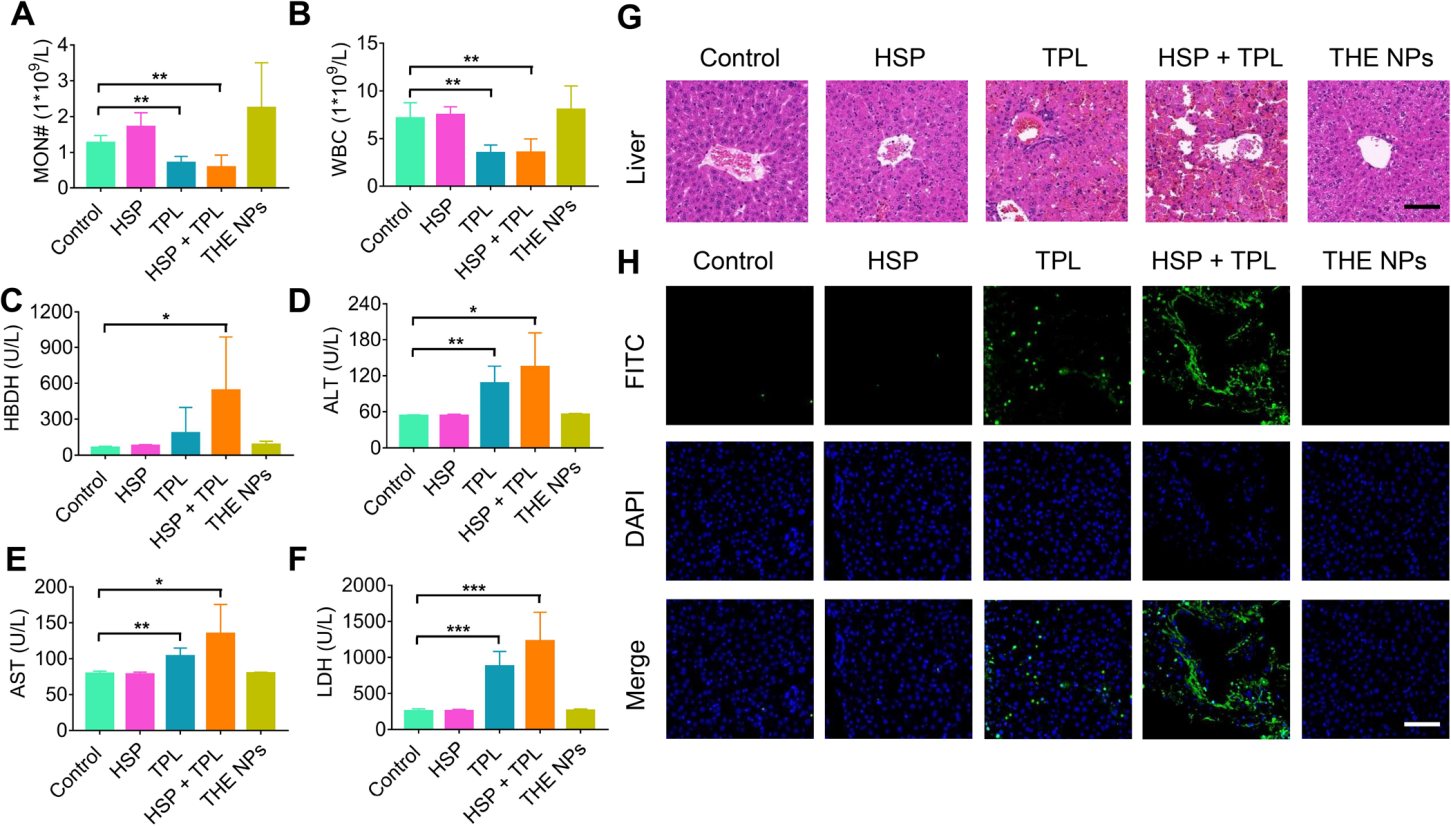
THE NPs has low toxicity in vivo. (A–H) The C57 mice were injected with *n* PBS, HSP (8.5 mg kg^‐1^), TPL (5 mg kg^‐1^), HSP (8.5 mg kg^‐1^) + TPL (5 mg kg^‐1^) and THE NPs (15 mg kg^‐1^) by tail vein for 24 h, and were subject to (A,B) blood indexes, (C–F) biochemical indexes, (G) HE, and (H) TUNEL analyses, *n* = 3. The scale bar is 50 µm. *p < 0.05; **p < 0.01; ***p < 0.001.

To investigate the biocompatibility of the THE NPs, C57 mice were used to test acute toxicity. Following TPL or HSP+TPL treatment, it was found that the number of monocytes (MON#) and white blood cells (WBCs) dramatically decreased, while the ALT, AST, HBDH, and LDH levels increased significantly (Figure 6A–F). No significant differences of TB, Cr, PK, UA, CK, TP, or ALB, were observed between the control and treatment groups (Figure S20). These results suggested that TPL causes serious damage to the blood, liver, and heart. However, there were no significant differences in these indices between the NP‐treated and control groups, thereby indicating that the NPs can effectively reduce the toxicity of TPL. Furthermore, it was observed that the ALP levels decreased significantly after treatment with HSP or the THE NPs (Figure S20). Since ALP is associated with liver failure, TUNEL and H&E (hematoxylin and eosin) staining were performed to analyze for apoptosis and injury in the mouse liver tissues after drug treatment. The results of the TUNEL assay showed that the apoptosis levels of the TPL and HSP+TPL treatment groups were significantly increased, but there were no significant differences between the HSP, THE NPs, and control groups (Figure 6H). Moreover, H&E staining of the livers demonstrated that in comparison to the control group, the livers of the mice treated with TPL or HSP+TPL were seriously injured, exhibiting congestion of the hepatic sinusoids, rupture of the hepatic plates, and necrosis of the hepatocytes (especially in the HSP+TPL treatment group); in contrast, these injuries were inappreciable in the HSP and NPs treatment groups (Figure 6G). Finally, the TUNEL detection results were found to be consistent with these morphological changes (Figure 6H). Therefore, these data demonstrated that the THE NPs not only exhibited an excellent antitumor effect, but that they also greatly reduced the biological toxicity of TPL.

## 4 CONCLUSION

In this work, bioinformatics analysis, molecular modeling, and surface plasmon resonance experiments were employed to demonstrate that triptolide (TPL) and hesperidin (HSP) possess a common target (i.e., the epidermal growth factor receptor, EGFR) in bladder cancer (BCa) cells, and that both drugs exhibit a high affinity for the EGFR. Therefore, HSP and TPL were conjugated into amphiphilic THE molecules by means of an ester bond and were subsequently assembled into nanoparticles (THE NPs) to achieve synergistic antitumor effects and improved biocompatibility. It was deduced that the NPs enter the tumor cells through endocytosis and are hydrolyzed into the individual HSP and TPL drug molecules under the acidic conditions of the lysosomes. As a result, HSP and TPL synergistically inhibited the activity of the EGFR/PI3K/AKT signaling pathway, thereby suppressing the proliferation of BCa cells whilst also promoting their apoptosis in vitro. In addition, the THE NPs effectively resolved the issues related to the poor water solubility, low bioavailability, and high toxicity of TPL. Furthermore, the developed NPs were demonstrated to effectively inhibit the proliferation of BCa cells in vivo. Overall, the obtained results indicate that the THE NPs can target the EGFR and inhibit its signaling and downstream pathways in the model of bladder cancer. Notably, the NP system preserved the bioactivities of TPL and HSP while lowering the overall toxicity compared to the individual drug molecules alone. The dual‐targeting function and high drug loading achieved using these NPs led to a significant antitumor effect, thereby providing a new option for BCa treatment.

## CONFLICT OF INTEREST STATEMENT

The authors declare no conflicts of interest.

## Supporting information

Supporting Information

## Data Availability

All data needed to evaluate the conclusions in the paper are present in the paper and/or in the Supporting Information. Additional data related to this paper may be requested from the authors.
