# Supplementary material for: Small‐molecule nanoprodrug with high drug loading and EGFR, PI3K/AKT dual‐inhibiting properties for bladder cancer treatment
Source: Exploration (Beijing). 2023 Jul 12;3(5):20220141. doi: 10.1002/EXP.20220141 (PMC10582605; doi:10.1002/EXP.20220141)
Supplement: Supplementary file 1 — Supporting Information [file EXP2-3-20220141-s001.pdf]

Supporting Information

Small-molecule nanoprodrug with high drug loading and EGFR, PI3K/AKT dual-inhibiting properties for bladder cancer treatment

Guoyin Li[1, 2, #], Zewen Song[3, #], Yi Ru[2], Jing Zhang[4], Lianxiang Luo[5], Wei Yang[6], Hao Wu[7], Haibao Jin[8], Xuanwen Bao[9], Di Wei[10], Zhao Yan[10], Haijing Qu[11], Zheng Zhu[12, *], Xiangdong Xue[11, *], Gang Zhou[13,*]

1. General information

Triptolide was purchased from various commercial sources including Tansoole and Meilunbio. General chemicals were purchased from Adamas-beta® and Energy Chemical in the highest purity and used without purification. All reactions and manipulations were performed using standard Schlenk techniques. CH_2Cl_2, $CHCl_3$ and ethyl acetate were distilled form CaH_2 under an atmosphere of argon. NMR spectra were recorded on a Bruker 400 MHz NMR spectrometer with TMS as the internal standard. HRMS were recorded on ZAB-HS spectrometer with ES ionization (ESI). HPLC analysis was performed on an Hanbo Sci&Tech NS4201 system with a NU3000 serials UV/VIS detector and two NP7000 serials pumps.

2. General procedure for the preparation of compound 1

Pyridin (5 mL) was added to a mixture of triptolide (1.08 g, 3 mmol) and succinic anhydride (4 g, 10 mmol), followed by DMAP (61 mg, 0.5 mmol). After stirring overnight, the mixture was diluted with ethyl acetate (20 mL), and then washed with saturated copper sulfate (3 x 30 mL), water and brine, respectively. The combined organic layers were dried over anhydrous sodium sulphate. Filtration followed by evaporation of the solvent under reduced pressure gave a residue. The residue was purified by silica gel column chromatography (Hexane/Ethyl acetate, 1:1) to give compound **1** (970 mg, 2.11 mmol, 70%) as a white solid.

3. General procedure for the preparation of compound 3

To a solution of hesperidin (6.1 g, 10 mmol) in pyridine (50 mL) were added acetic anhydride (50 mL). After stirring for 24 h at 90 °C, the reaction mixture was cooled to

room temperature and the solvent was evaporated under vacuum. The residue was poured into water, the precipitates were collected by filtration, and then dissolved in chloroform. The mixture was filtered through a pad of celite to remove insoluble materials. The combined filtrate and washings were dried over anhydrous sodium sulphate. Filtration followed by evaporation of the solvent under reduced pressure gave a residue. The residue was purified by silica gel column chromatography (Hexane/Ethyl acetate, 1:3) to give compound **3** (7.1 g, 7.51 mmol, 75%) as a white amorphous solid.

4. General procedure for the preparation of catalyst 5

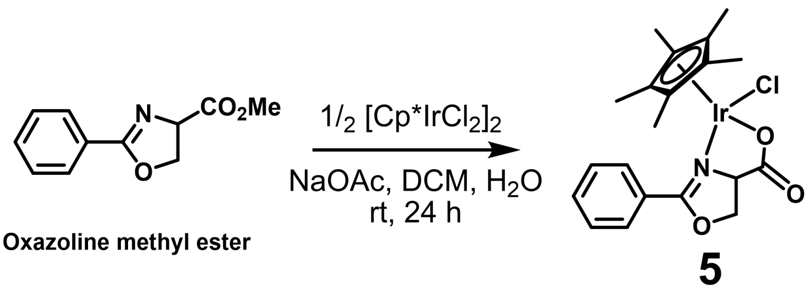

Oxazoline methyl ester (100 mg, 0.49 mmol), $[Cp^*IrCl_2]_2$ (170 mg, 0.22 mmol), NaOAc (402 mg, 4.9 mmol) were placed in a Schlenk tube. DCM (10 mL) and H_2O (0.2 mL) were introduced and the resulting mixture was stirred for 24 h at room temperature. The reaction mixture was then filtered through celite and dried over Na_2SO_4. The solvent was evaporated under vacuum and the resulting solid was washed with diethyl ether/hexane to remove excess ligand.

5. General procedure for the preparation of compound 4

Compound **3** (2.0 g), catalyst **5** (1.3 mg) were placed in a Schlenk tube. DCM (5 mL) was introduced with a syringe and the resulting mixture was bubbled with nitrogen for 2 min. The tube was then degassed and recharged with nitrogen three times. The F/T azeotrope (1 mL) was then added and the mixture was stirred at room temperature overnight. The reaction mixture was washed with water and dried over anhydrous sodium sulphate. The solvent was then removed under reduced pressure

and flash column chromatography was applied to obtain the pure product **4** (1.6 g, 1.68 mmol, 79%) as a light yellow amorphous solid.

6. General procedure for the preparation of THE

To a solution of compound **1** (970 mg, 2.11 mmol) in dry DCM (10 mL), an excess of oxalyl chloride (5 mL) was added at 0 °C, continue stirring for 4 h at room temperature, the oxalyl chloride was removed by rotary evaporation and an oil pump was used for 30 minutes to ensure that the oxalyl chloride was completely removed. The obtained solid was added to dry DCM (5 ml) to dissolve it, then added dropwise to a solution of compound **4** (1.9 g, 2.0 mmol) in dry DCM (10 mL) at 0 °C, after returning to room temperature, the mixture was stirred overnight. The solvent was evaporated under vacuum and the obtained solid continued to the next step without further purification. To a magnetically stirred solution of last step product in MeOH (10 mL), was added acetyl chloride (20 μL, 0.3 mmol) at room temperature. The mixture was stirred for 3 h, the color of the solution turns yellow, the solvent was then removed under reduced pressure and flash column chromatography (Methanol/Ethyl acetate, 1:5) was applied to obtain the pure product THE (1.3 g, 1.23 mmol, 62%) as a yellow solid.

7. Analytic data of products

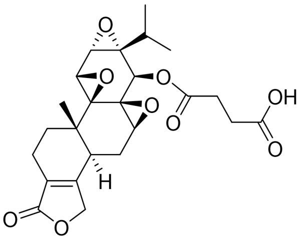

compound 1:

70% yield, white solid. ^{1}H NMR (400 MHz, $CDCl_3$) δ 5.11 (s, 1H), 4.69 (s, 2H), 3.85 (d, *J* = 3.1 Hz, 1H), 3.55 (d, *J* = 3.1 Hz, 1H), 3.48 (d, *J* = 5.5 Hz, 1H), 2.88 – 2.66 (m, 5H), 2.34 (d, *J* = 18.5 Hz, 1H), 2.19 (dt, *J* = 15.2, 5.6 Hz, 2H), 1.98 – 1.86 (m, 2H), 1.59 (dd, *J* = 12.5, 5.2 Hz, 1H), 1.32 – 1.19 (m, 2H), 1.07 (s, 3H), 0.97 (d, *J* = 7.0, 3H), 0.86 (d, *J* = 7.0, 3H). ^{13}C NMR (101 MHz, $CDCl_3$) δ 177.45, 173.38,

171.60, 160.23, 125.49, 71.37, 70.05, 63.57, 63.30, 61.17, 59.62, 55.35, 55.00, 40.32, 35.65, 29.79, 28.94, 28.81, 28.04, 23.37, 17.44, 17.02, 16.65, 13.69.

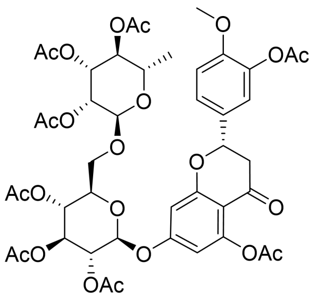

compound 3:

75% yield, white amorphous solid. ^{1}H NMR (400 MHz, $CDCl_3$) δ 7.36 (d, *J* = 8.8 Hz, 1H), 7.16 (d, *J* = 2.3 Hz, 1H), 7.00 (d, *J* = 8.5 Hz, 1H), 6.47 (d, *J* = 2.5 Hz, 1H), 6.31 (d, *J* = 2.4 Hz, 1H), 5.48 – 5.37 (m, 1H), 5.25 (d, *J* = 16.1 Hz, 6H), 5.02 (t, *J* = 10.2 Hz, 1H), 4.69 (s, 1H), 3.92 – 3.90 (m, 1H), 3.86 (s, 3H), 3.81 (dt, *J* = 11.7, 3.3 Hz, 2H), 3.64 (td, *J* = 11.6, 10.4, 4.9 Hz, 1H), 2.99 (dd, *J* = 16.6, 12.9 Hz, 1H), 2.76 (dd, *J* = 16.6, 3.0 Hz, 1H), 2.38 (s, 3H), 2.33 (s, 3H), 2.09 (s, 3H), 2.08 (s, 3H), 2.04 (s, 9H), 1.96 (s, 3H), 1.15 (d, *J* = 6.3 Hz, 3H). ^{13}C NMR (101 MHz, $CDCl_3$) δ 188.73, 170.19, 170.03, 169.85, 169.34, 169.15, 168.87, 163.80, 161.89, 151.89, 151.59, 139.93, 130.74, 124.95, 121.18, 112.45, 109.60, 105.91, 102.16, 98.09, 97.59, 78.68, 77.22, 73.26, 72.45, 70.87, 70.80, 69.36, 68.97, 68.61, 66.66, 66.15, 56.04, 21.05, 20.80, 20.66, 20.63, 20.60, 17.28. HRMS for $C_{44}H_{50}O_{23}$ $[M+H]^+$: m/z calc., 946.2723; found, 946.2743.

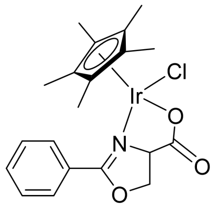

catalyst 5:

62% yield, yellow solid. ^{1}H NMR (400 MHz, $CDCl_3$) δ 8.38 (d, *J* = 7.6 Hz, 2H), 7.62 – 7.56 (m, 1H), 7.49 – 7.42 (m, 2H), 4.88 (dd, *J* = 11.0, 8.2 Hz, 1H), 4.84 – 4.75 (m, 1H), 4.51 (dd, *J* = 11.9, 8.1 Hz, 1H), 1.64 (s, 15H). ^{13}C NMR (101 MHz, $CDCl_3$) δ 175.01, 167.38, 133.32, 129.99, 128.27, 124.14, 85.15, 72.05, 71.28, 9.21. HRMS for $C_{20}H_{23}ClIrNO_3$ $[M+Na]^+$: m/z calc., 576.0894; found, 576.0889.

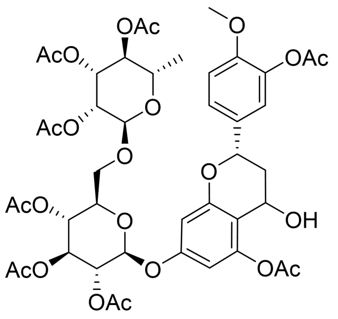

compound 4:

79% yield, light yellow amorphous solid. ^{1}H NMR (400 MHz, $CDCl_3$) δ 7.33 – 7.28 (m, 1H), 7.16 (t, *J* = 2.3 Hz, 1H), 7.05 – 6.97 (m, 1H), 6.48 (d, *J* = 2.5 Hz, 1H), 6.32 (d, *J* = 2.4 Hz, 1H), 5.49 – 5.35 (m, 1H), 5.30 – 5.10 (m, 7H), 5.02 (t, *J* = 10.0 Hz, 1H), 4.70 (s, 1H), 3.95 – 3.87 (m, 1H), 3.86 (s, 3H), 3.85 – 3.75 (m, 1H), 3.64 (td, *J* = 11.6, 10.4, 4.9 Hz, 1H), 3.69 – 3.58(m, 1H), 3.11 – 2.92 (m, 1H), 2.79 – 2.66 (m, 1H), 2.38 (s, 3H), 2.35 (s, 3H), 2.09 (s, 3H), 2.08 (s, 3H), 2.04 (s, 9H), 1.96 (s, 3H), 1.15 (d, *J* = 6.2 Hz, 3H). ^{13}C NMR (101 MHz, $CDCl_3$) δ 170.17, 170.01, 169.77, 169.44, 169.33, 169.14, 168.85, 163.98, 163.80, 161.88, 151.88, 151.58, 139.93, 130.74, 124.94, 121.18, 112.45, 109.60, 105.90, 102.16, 98.08, 97.58, 78.67, 73.26, 72.44, 70.87, 70.80, 69.35, 68.97, 68.63, 66.66, 66.14, 56.03, 53.81, 21.04, 20.79, 20.75, 20.64, 20.61, 20.59, 17.27. HRMS for $C_{44}H_{52}O_{23}$ $[M+H]^{+}$: m/z calc., 948.2919; found, 948.2932.

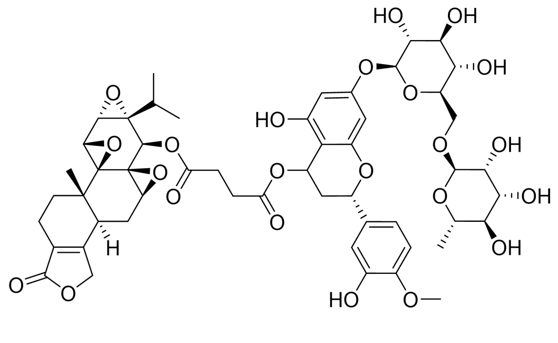

THE:

62% yield, yellow solid. ^{1}H NMR (400 MHz, CD_3OD) δ 7.54 (dd, *J* = 8.5, 2.1 Hz, 1H), 7.35 (d, *J* = 2.1 Hz, 1H), 7.00 (d, *J* = 8.6 Hz, 1H), 6.59 (d, *J* = 2.2 Hz, 1H), 6.44 (d, *J* = 2.2 Hz, 1H), 5.39 – 5.11 (m, 5H), 5.08 (s, 1H), 5.02 (t, *J* = 9.6 Hz, 1H), 4.73 (s, 1H), 4.68 (s, 2H), 3.97 (s, 3H), 3.88 – 3.80 (m, 3H), 3.79 – 3.63 (m, 1H), 3.54 (d, *J* =

3.0 Hz, 1H), 3.46 (d, *J* = 5.6 Hz, 1H), 2.90 – 2.59 (m, 6H), 2.38 – 2.24 (m, 2H), 2.23 – 2.11 (m, 3H), 1.93 – 1.85 (m, 2H), 1.57 (dd, *J* = 12.6, 5.2 Hz, 1H), 1.15 (d, *J* = 6.3 Hz, 3H), 1.05 (s, 3H), 0.95 (d, *J* = 7.0 Hz, 3H), 0.83 (d, *J* = 6.9 Hz, 3H).

^{13}C NMR (101 MHz, CD_3OD) δ 182.48, 176.65, 164.51, 162.37, 161.87, 160.05, 157.34, 152.47, 149.35, 125.57, 123.54, 120.31, 111.24, 108.91, 106.84, 104.67, 99.62, 98.10, 95.54, 73.40, 72.49, 71.34, 71.04, 70.93, 70.00, 69.47, 68.91, 68.72, 66.70, 63.55, 63.30, 61.15, 59.63, 56.14, 56.12, 55.36, 54.99, 40.35, 35.66, 29.81, 29.00, 28.75, 28.04, 23.40, 20.77, 20.63, 20.60, 17.45, 17.29, 17.04, 16.65, 13.69.

HRMS for $C_{52}H_{62}O_{23}$ $[M+H]^+$: m/z calc., 1054.3761; found, 1054.3787.

8. SPR

LMW Kinetics-EGFR & H

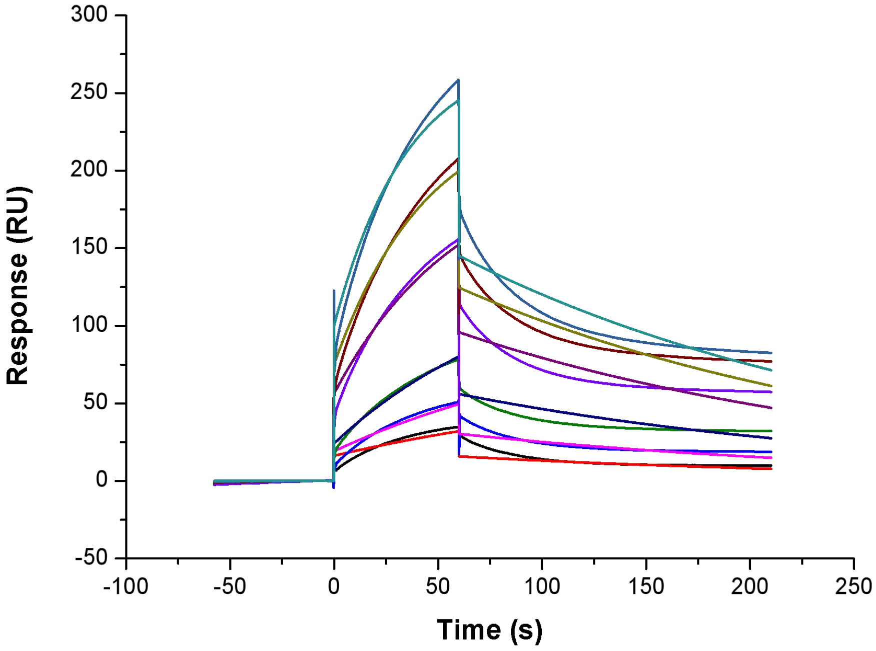

Curve	ka (1/Ms)	kd (1/s)	KD (M)	Rmax (RU)	Conc (M)	kt (RU/Ms)	RI (RU)	Chi² (RU²)	U-value
	29.82	0.004729	1.59E-04	212				31.9	2
Cycle: 6 50 µM					5.00E-05	1.34E+11	16.24		
Cycle: 7 100 µM					1.00E-04	1.34E+11	19.19		
Cycle: 8 200 µM					2.00E-04	1.34E+11	24.21		
Cycle: 9 400 µM					4.00E-04	1.34E+11	56.46		
Cycle: 10 600 µM					6.00E-04	1.34E+11	75.28		

LMW Kinetics-EGFR & T

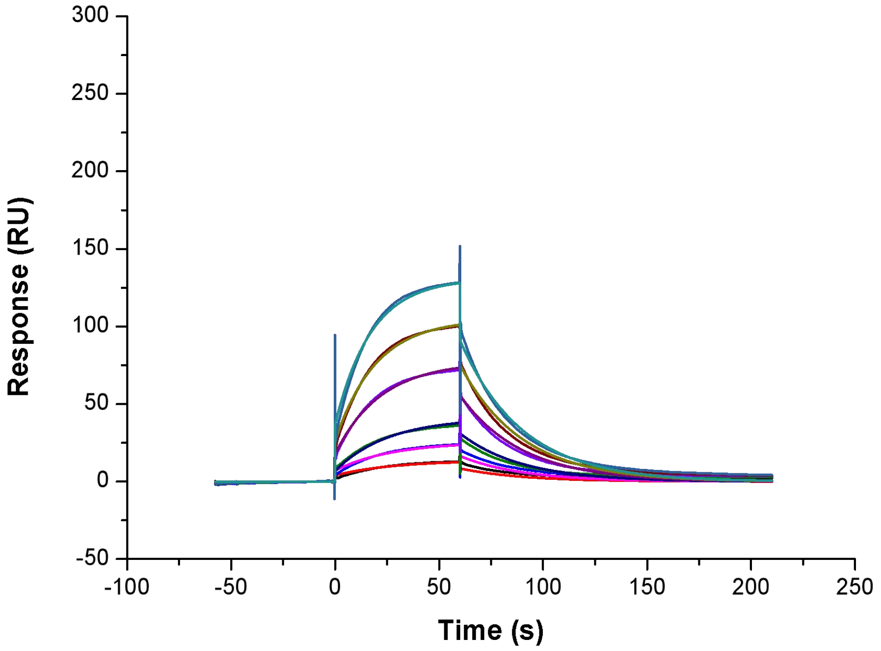

Curve	ka (1/Ms)	kd (1/s)	KD (M)	Rmax (RU)	Conc (M)	kt (RU/Ms)	RI (RU)	Chi² (RU²)	U-value
	30.18	0.03361	0.001114	224.2				5.19	3
Cycle: 5 50 µM					5.00E-05	2.48E+10	4.005		
Cycle: 6 100 µM					1.00E-04	2.48E+10	7.137		
Cycle: 7 200 µM					2.00E-04	2.48E+10	6.844		
Cycle: 8 400 µM					4.00E-04	2.48E+10	17.96		
Cycle: 9 600 µM					6.00E-04	2.48E+10	26.2		
Cycle: 10 800 µM					8.00E-04	2.48E+10	37.25		

LMW Kinetics-EGFR & T+H

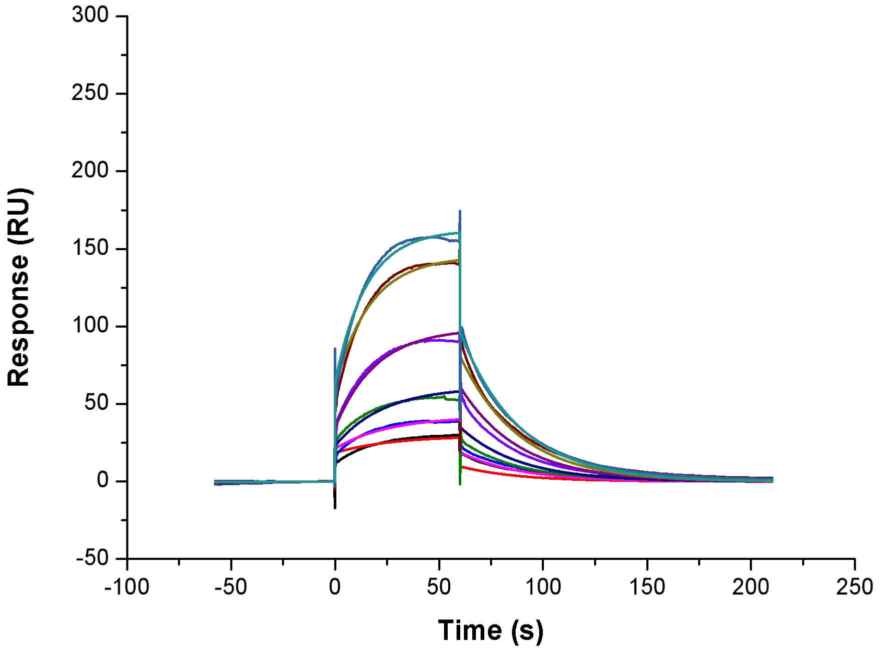

Curve		ka (1/Ms)	kd (1/s)	KD (M)	Rmax (RU)	Conc (M)	kt (RU/Ms)	RI (RU)	Chi² (RU²)	U-value
		39.32	0.0353	8.98E-04	207.8				7.58	3
Cycle: 5	50 µM					5.00E-05	1.47E+11	18.49		
Cycle: 6	100 µM					1.00E-04	1.47E+11	21.23		
Cycle: 7	200 µM					2.00E-04	1.47E+11	23.16		
Cycle: 8	400 µM					4.00E-04	1.47E+11	34.81		
Cycle: 9	600 µM					6.00E-04	1.47E+11	62		
Cycle: 10	800 µM					8.00E-04	1.47E+11	64.35		

LMW Kinetics-EGFR & Nano

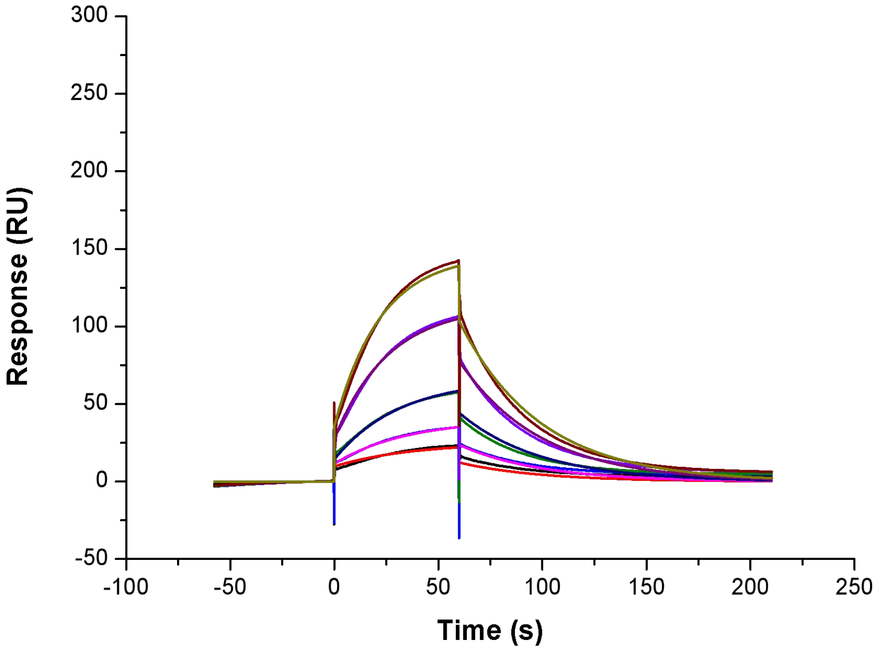

Curve	ka (1/Ms)	kd (1/s)	KD (M)	Rmax (RU)	Conc (M)	kt (RU/Ms)	RI (RU)	Chi² (RU²)	U-value
	32.55	0.02567	7.89E-04	255.5				9.63	4
Cycle: 6 50 µM					5.00E-05	7.24E+09	9.77		
Cycle: 7 100 µM					1.00E-04	7.24E+09	11.49		
Cycle: 8 200 µM					2.00E-04	7.24E+09	14.36		
Cycle: 9 400 µM					4.00E-04	7.24E+09	27.6		
Cycle: 10 600 µM					6.00E-04	7.24E+09	36.08		

9. Supplementary figures

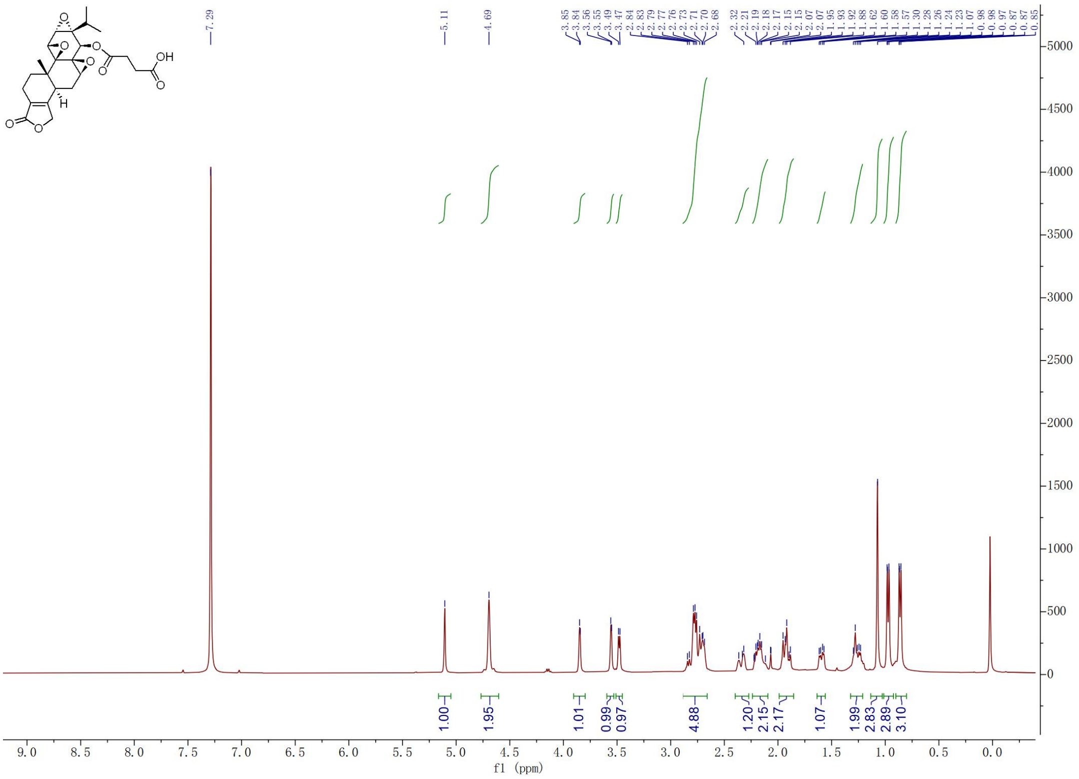

Figure S1. ^{1}H NMR spectrum of compound **1** in $CDCl_3$.

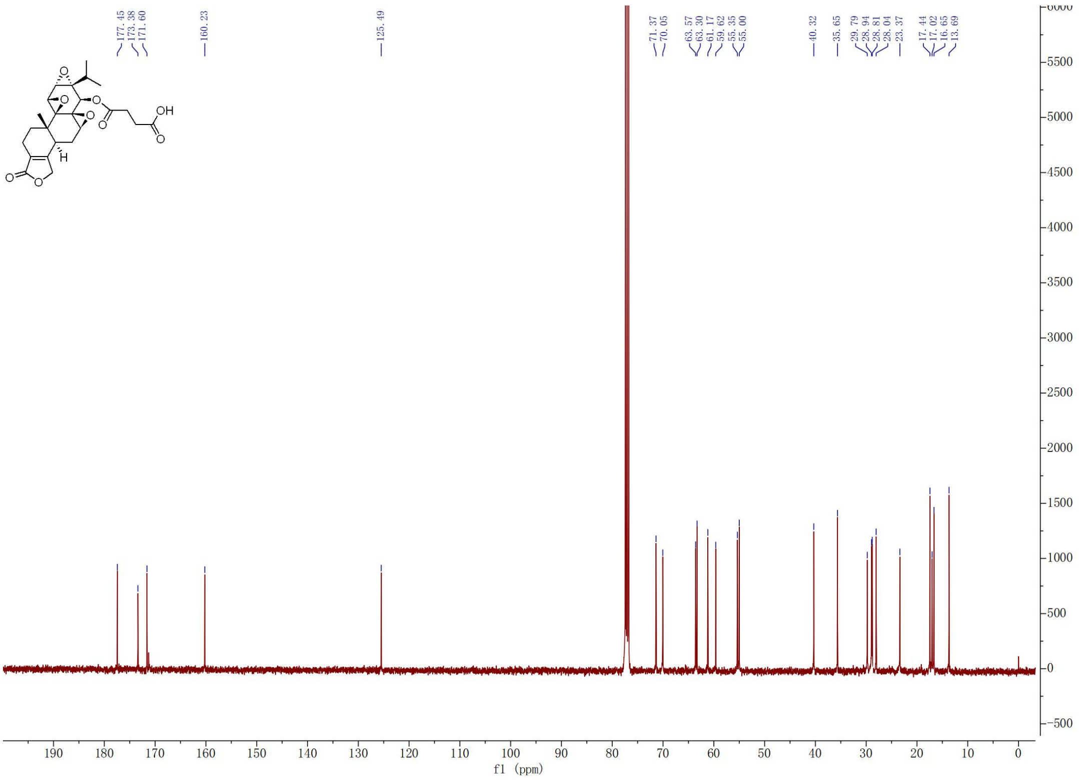

Figure S2. ^{13}C NMR spectrum of compound **1** in $CDCl_3$.

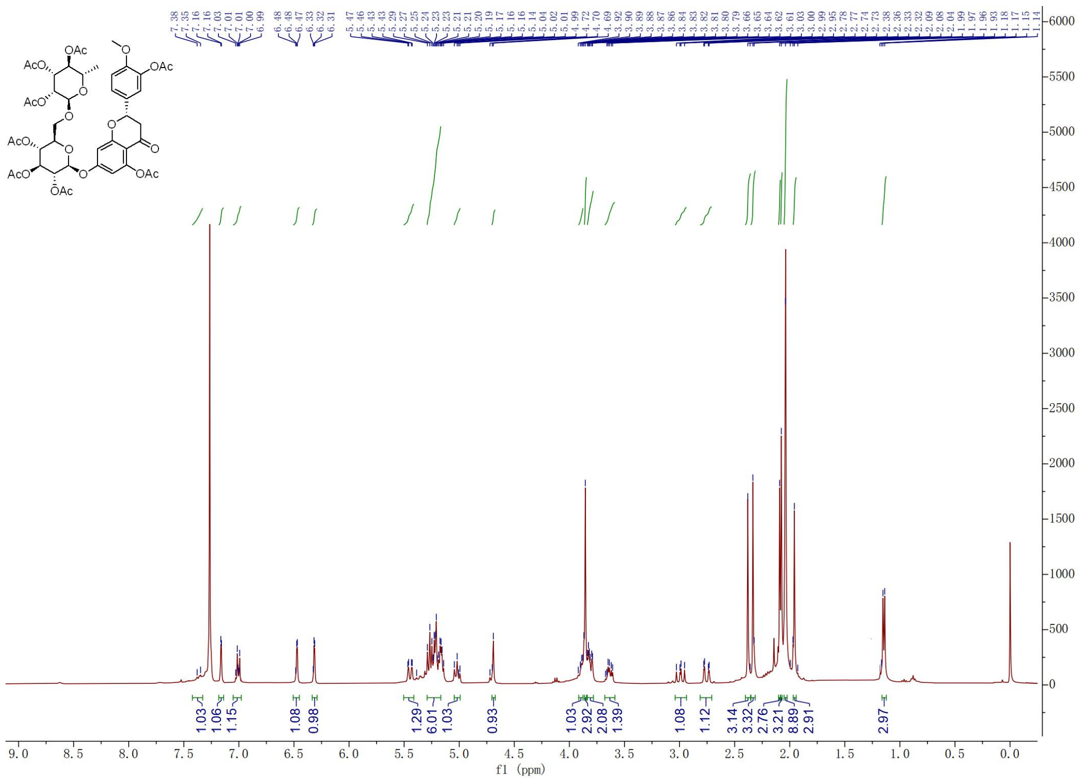

Figure S3. ^{1}H NMR spectrum of compound **3** in $CDCl_3$.

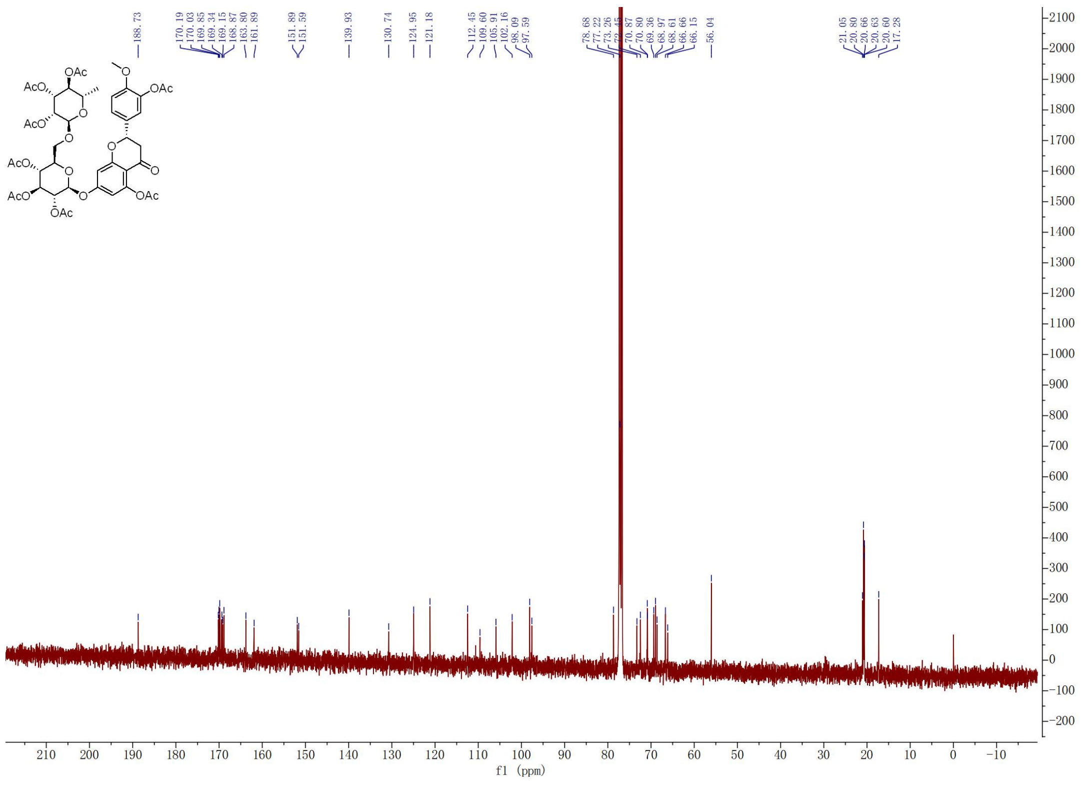

Figure S4. ^{13}C NMR spectrum of compound **3** in $CDCl_3$.

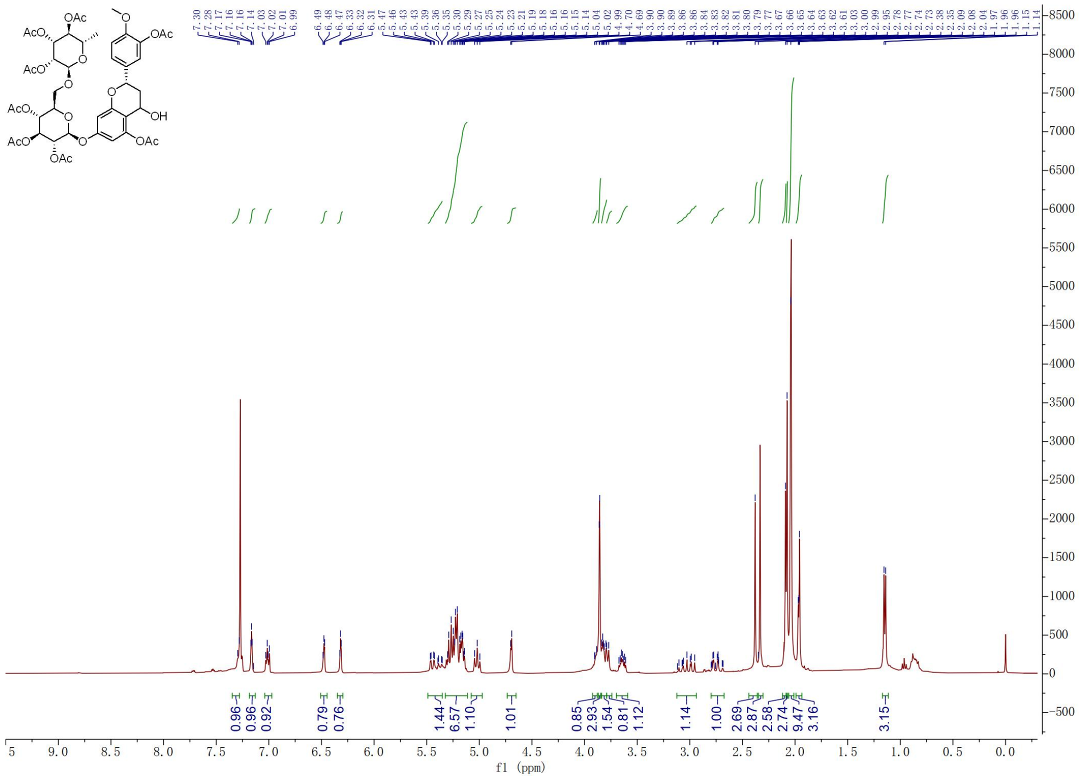

Figure S5. ^{1}H NMR spectrum of compound **4** in $CDCl_3$.

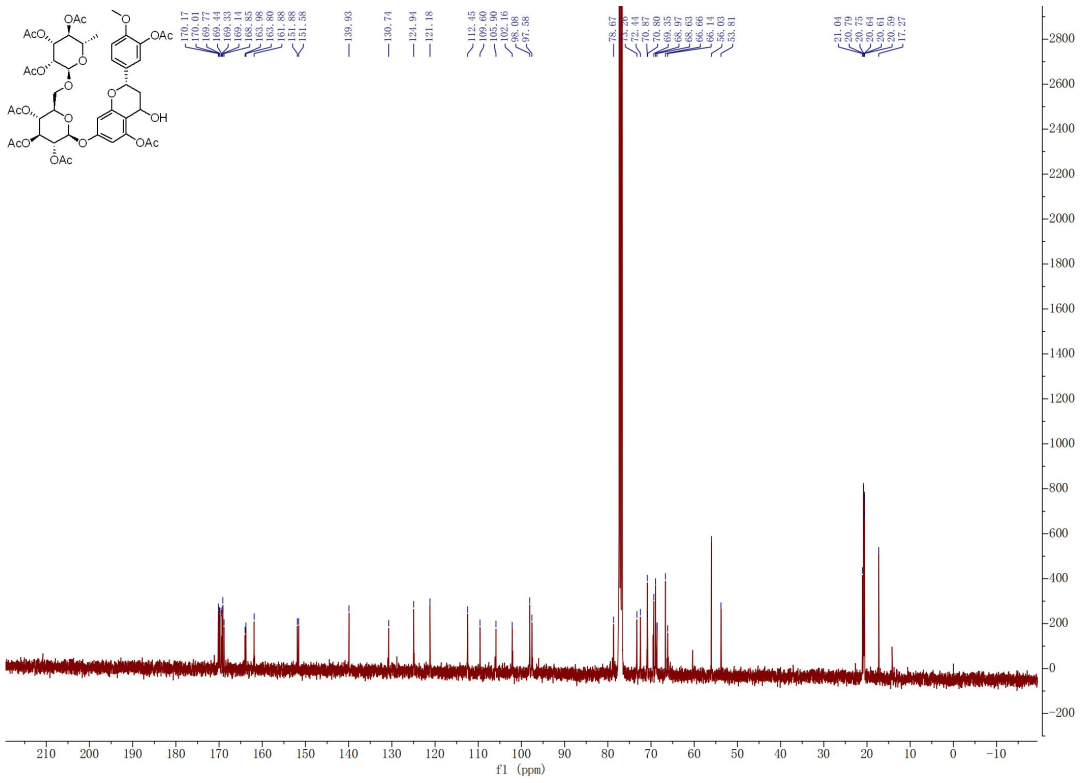

Figure S6. ^{13}C NMR spectrum of compound **4** in $CDCl_3$.

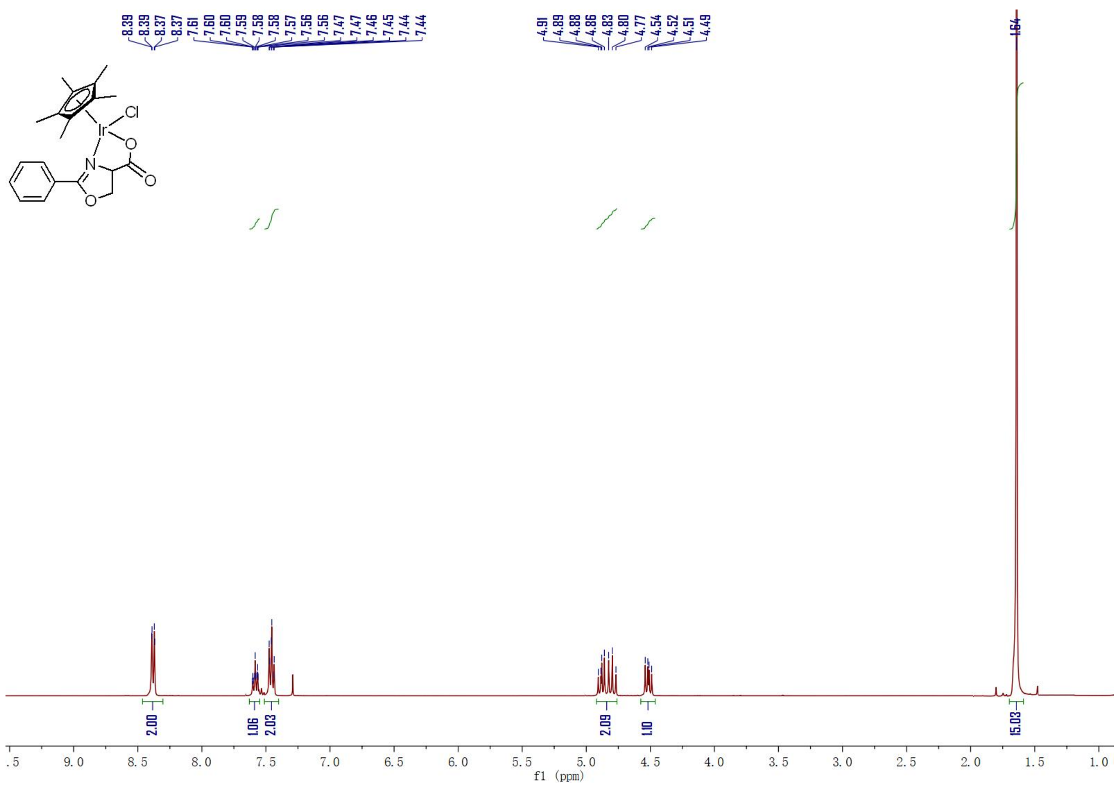

Figure S7. ^{1}H NMR spectrum of compound **5** in $CDCl_3$.

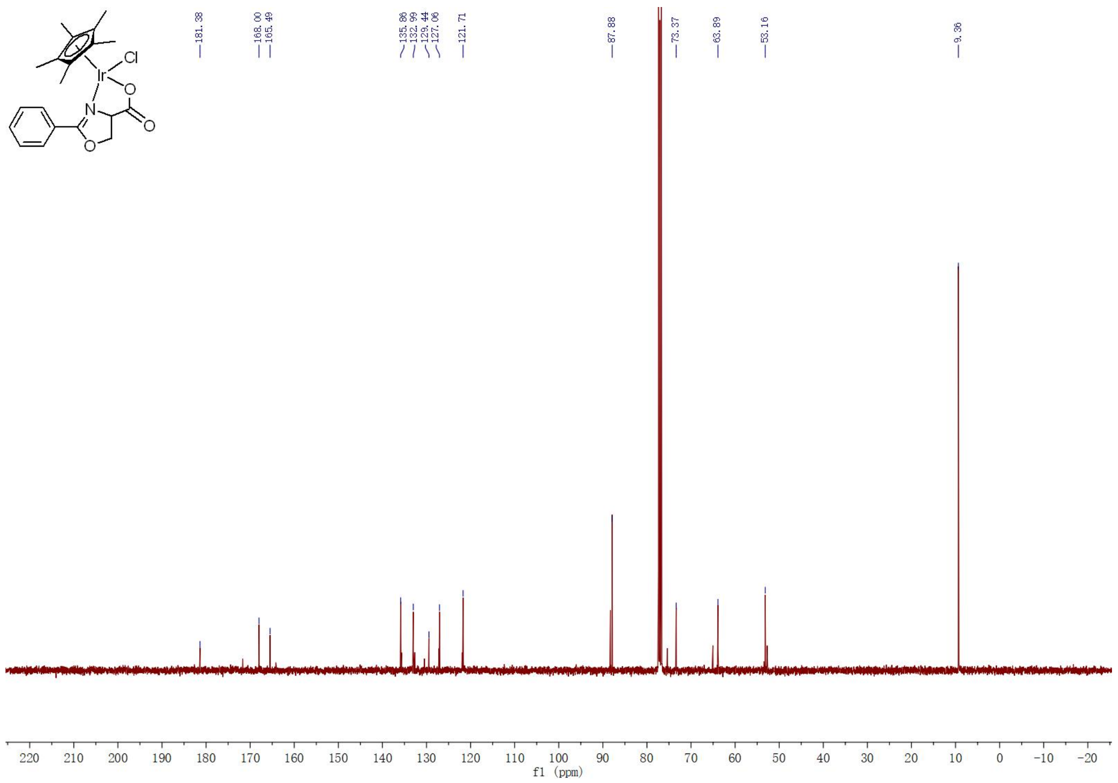

Figure S8. ^{13}C NMR spectrum of compound **5** in $CDCl_3$.

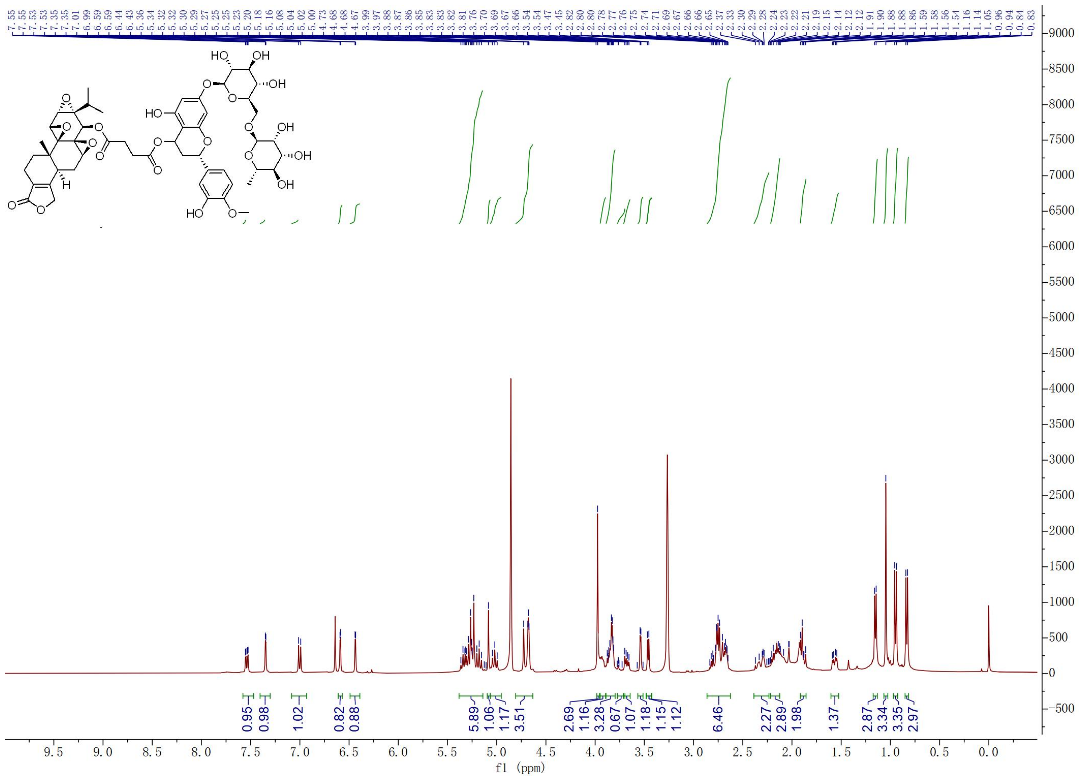

Figure S9. ^{1}H NMR spectrum of THE in CD_3OD.

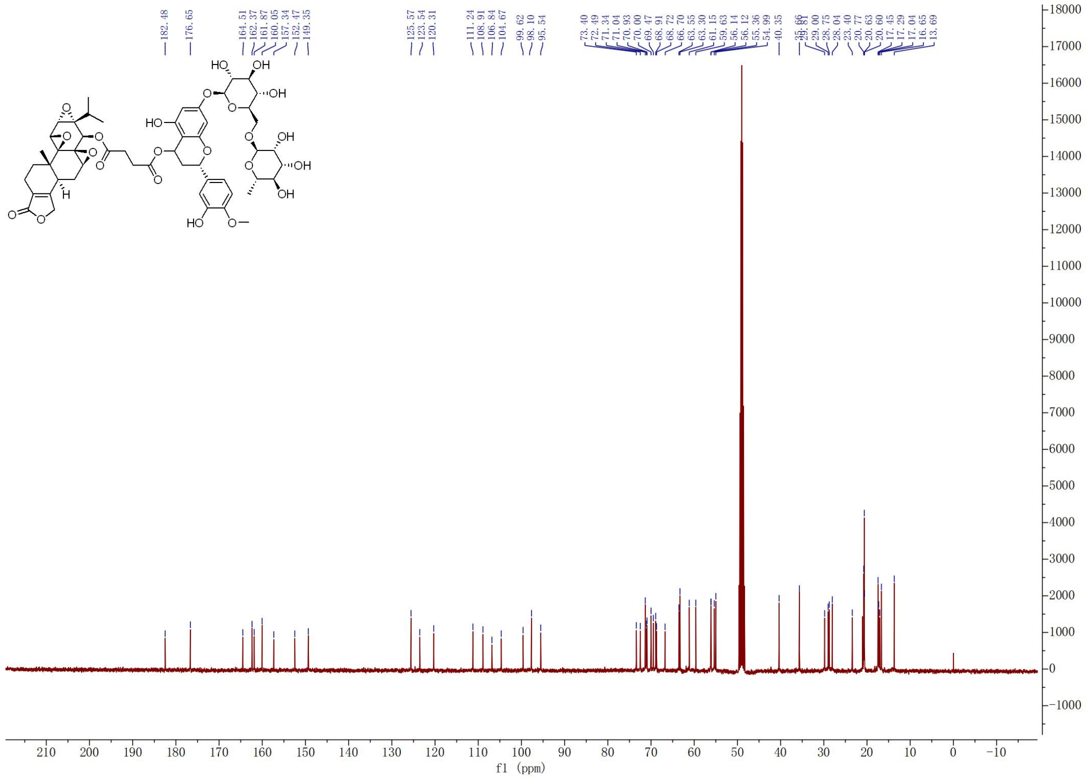

Figure S10. ^{13}C NMR spectrum of THE in CD_3OD.

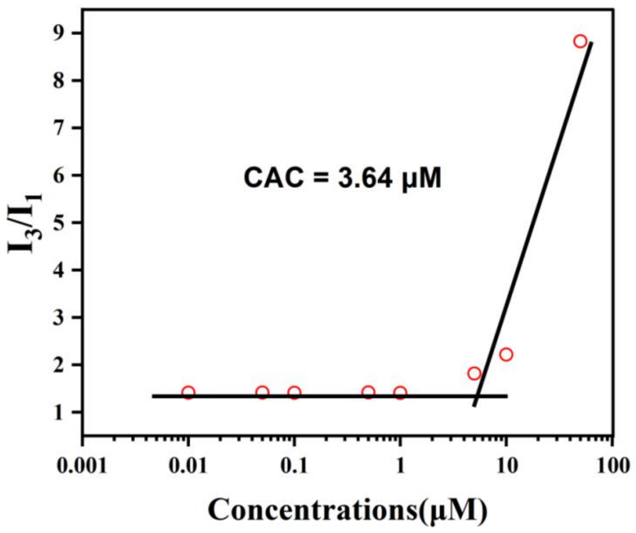

Figure S11. CAC value of THE NPs was detected via pyrene radiometric method

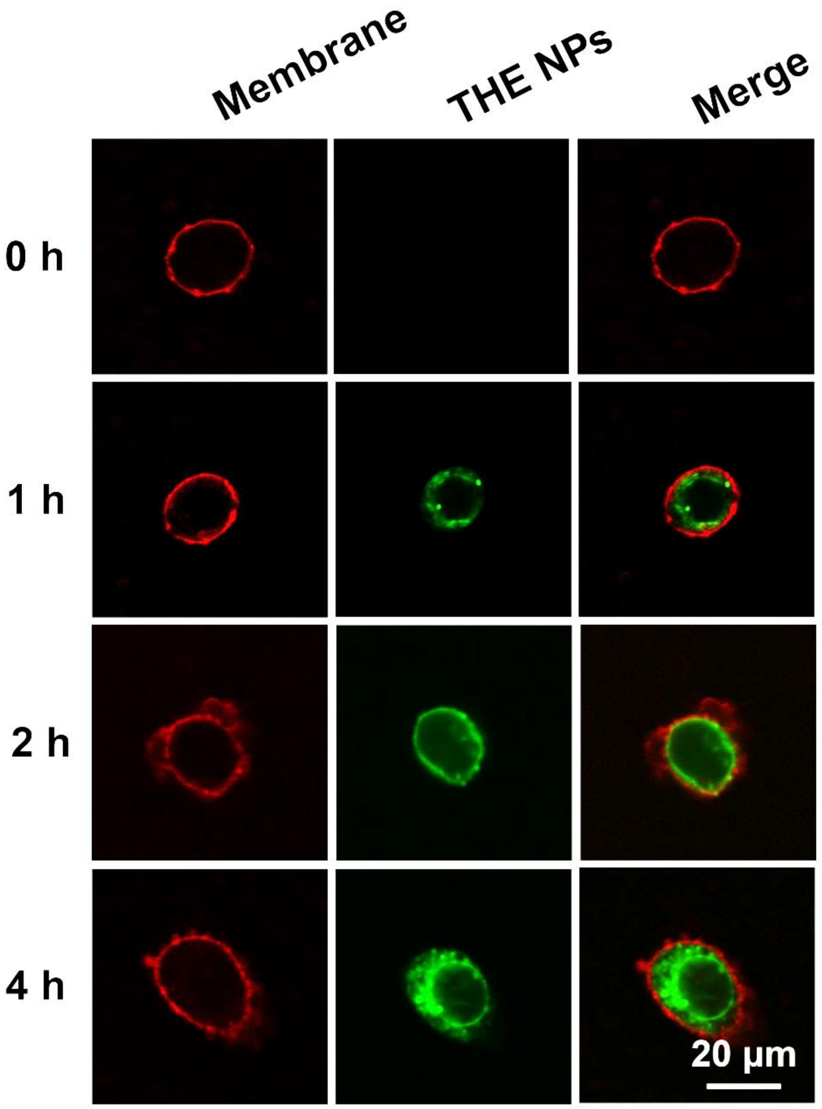

Figure S12. Distribution of THE NPs in 5637 cells: The distribution of THE NPs in 5637 cells at different time points using laser confocal microscopy. Cell membrane was stained with Dil showed red color. THE NPs showed green color as indicated by DiD.

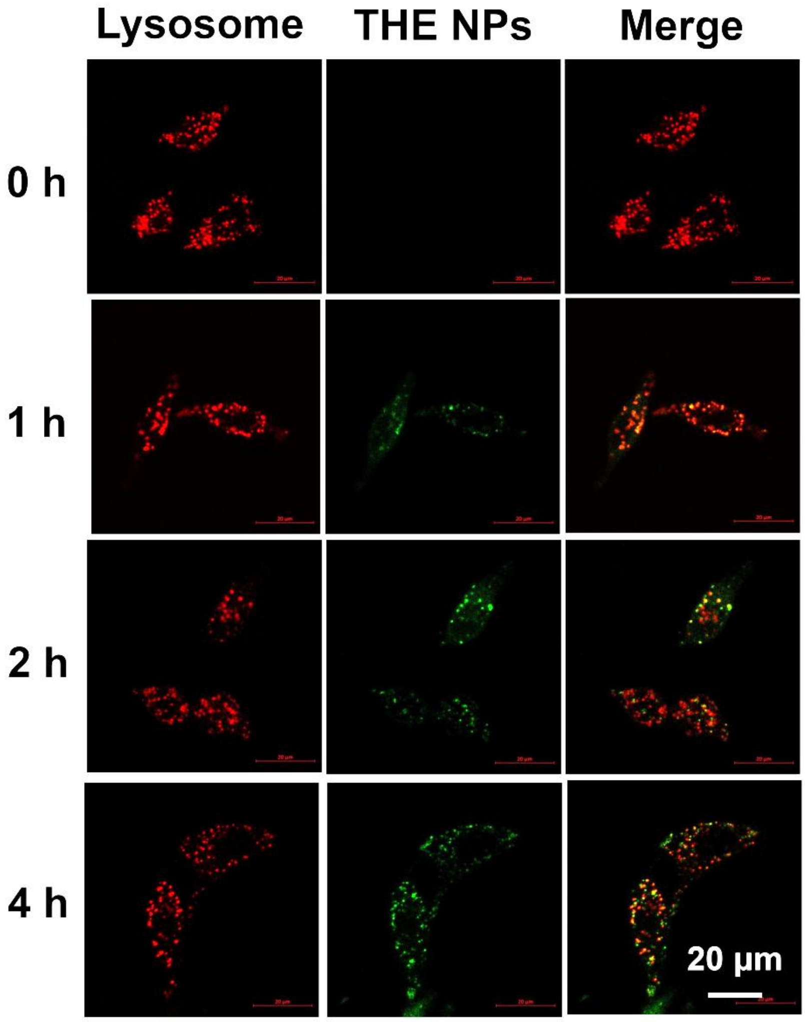

Figure S13. Distribution of THE NPs in lysosomes: The distribution of THE NPs in lysosomes at different time points were detected using laser confocal microscopy. The lysosomes of 5637 cells were stained with Lyso-Tracker showed red color. THE NPs showed green color as indicated by DiD.

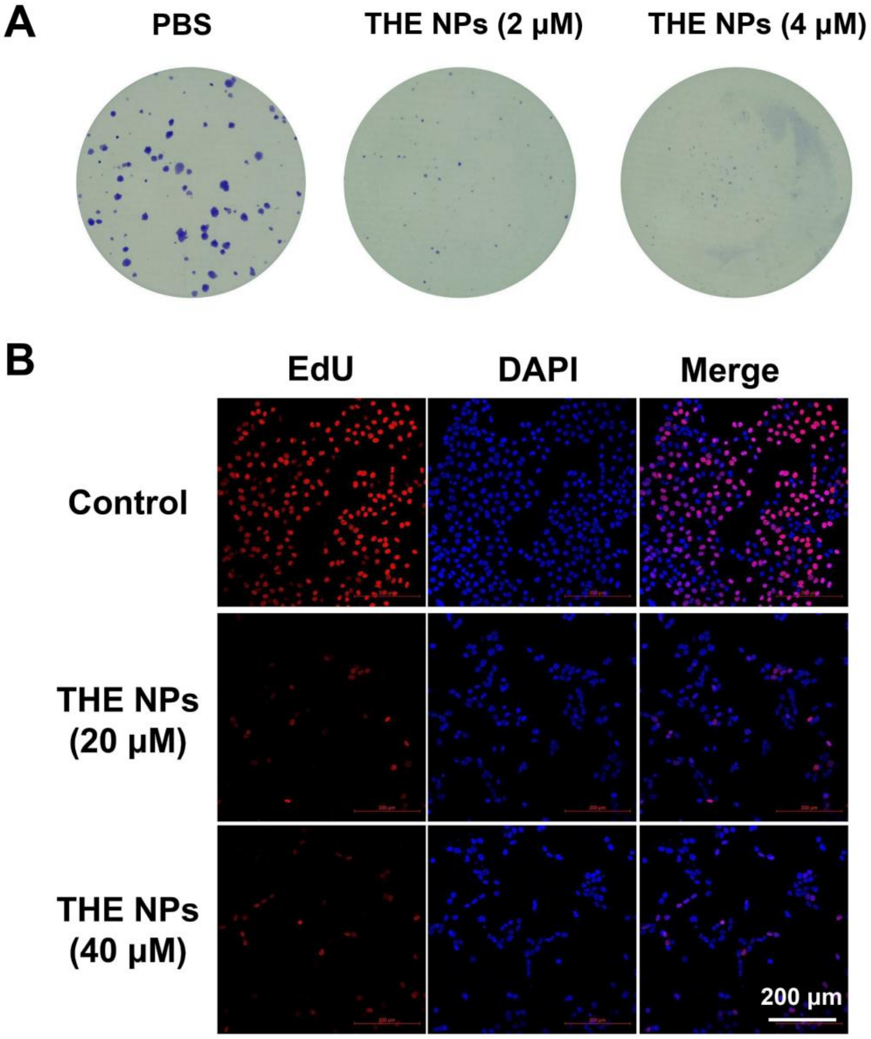

Figure S14. THE NPs can effectively inhibit bladder cancer cell proliferation

(A) The 5637 cells were treated with indicated dose of THE NPs for 14 day, and were stained with crystal violet. (B) The 5637 cells were treated with indicated dose of THE NPs for 24 h, and were subject to EdU stain. Proliferating 5637 cells positively stained with EdU showed red color. Cell nuclei stained with DAPI showed blue color.

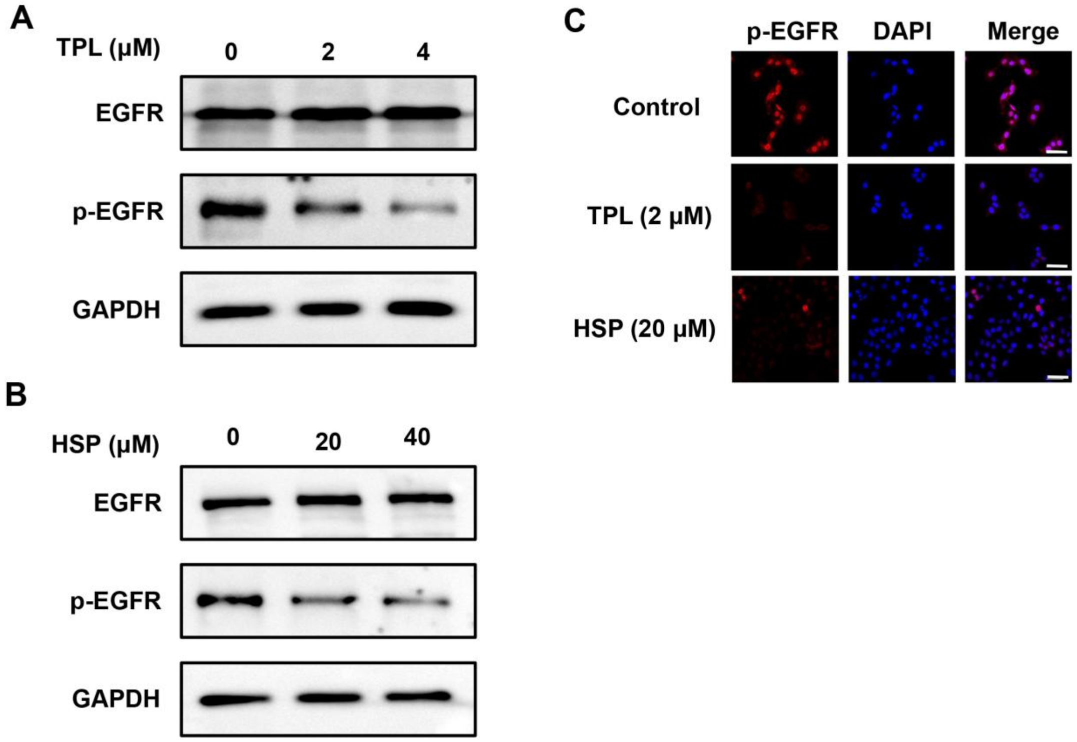

Figure S15. Both HSP and TPL can effectively inhibit EGFR phosphorylation in bladder cancer

(A) The 5637 cells were treated with indicated dose of TPL for 48 h, and were subject to Western blot analyses. (B) The 5637 cells were treated with indicated dose of HSP for 24 h, and were subject to Western blot analyses.The scale bar is 50 μm. (C) The 5637 cells were treated with indicated dose of TPL and HSP for 24 h, and were subject to ICC analyses. The scale bar is 50 μm.

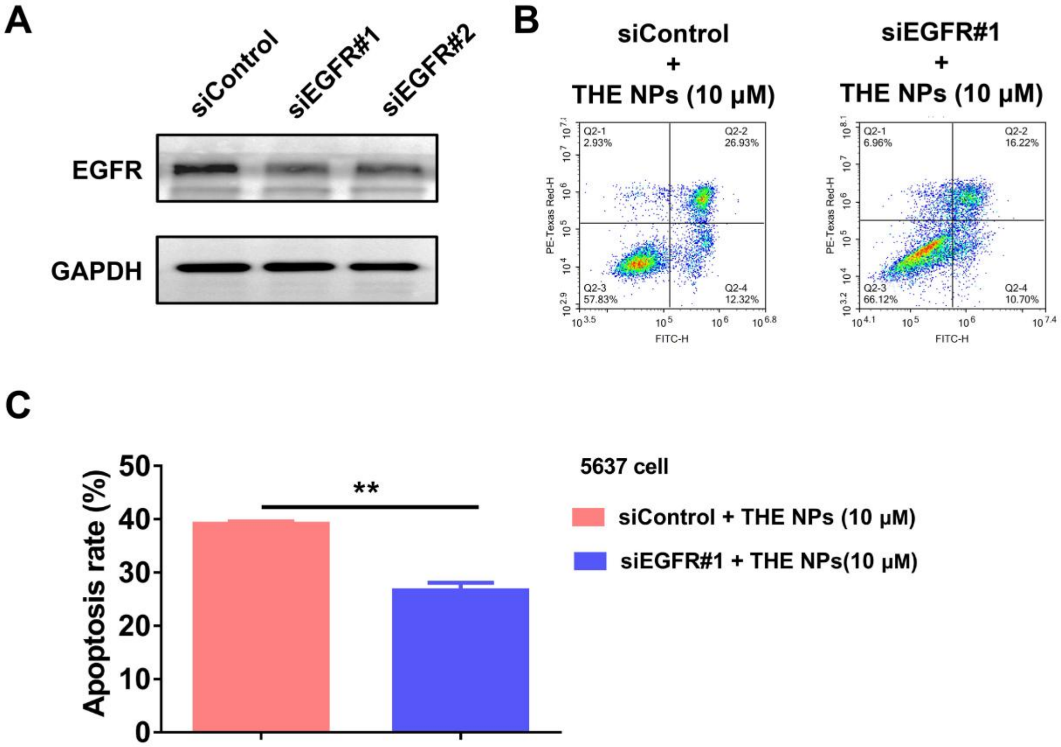

Figure S16. The killing effect of THE NPs on bladder cancer was weaken after knocking down EGFR

(A) The 5637 cells were transfected with siRNA for 48 h, and were subject to Western blot analyses. (B&C) The 5637 cells were transfected with siRNA for 48 h, and then were treated with THE NPs (10 μM) for 24 h, and were subject to flow cytometry assay for apoptosis. *, $p < 0.05$; **, $p < 0.01$; ***, $p < 0.001$

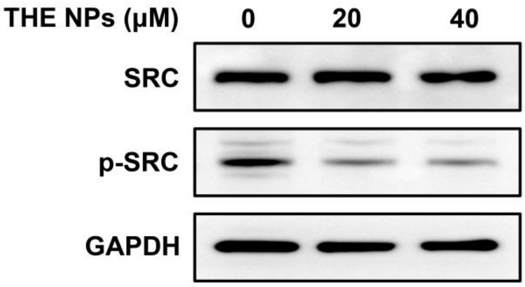

Figure S17. THE NPs can effectively inhibit SRC phosphorylation in bladder cancer: The 5637 cells were treated with indicated dose of THE NPs for 24 h, and were subject to Western blot analyses.

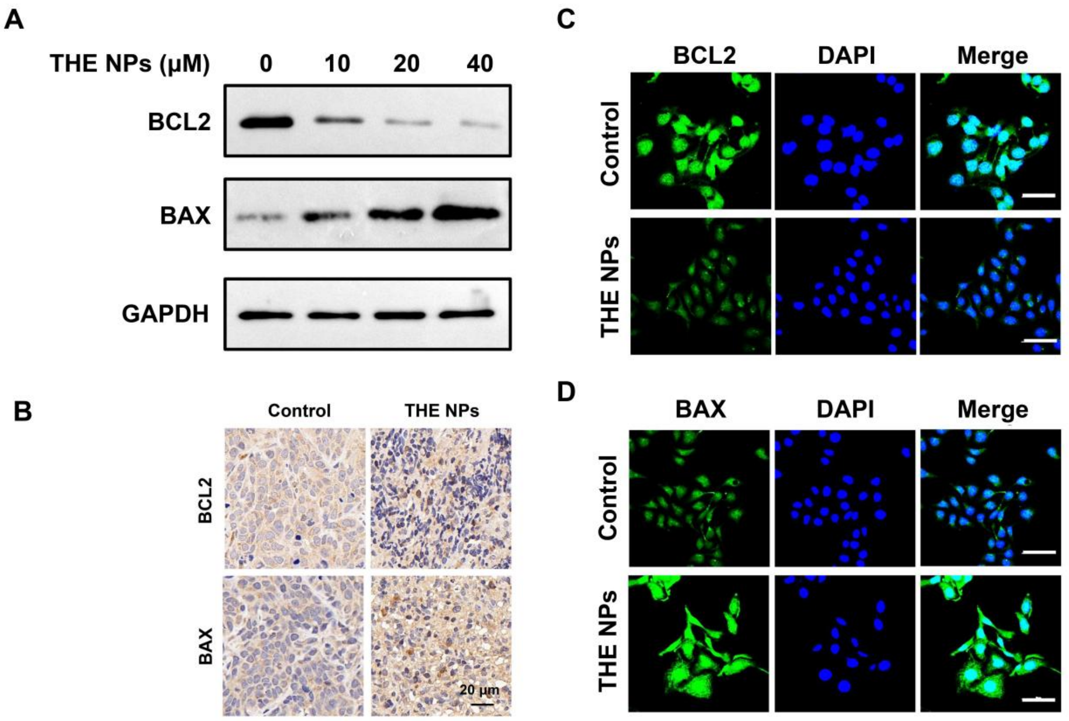

Figure S18. THE NPs can inhibit BCL2 and promote BAX expression

(A) The 5637 cells were treated with indicated dose of THE NPs for 24 h, and were subject to Western blot analyses. (B) Immunohistochemical analysis of BCL2 and BAXof cancer tissues from xenograft BCa models after 20 days of treatment with PBS and THE NPs. The scale bar is 20 μm. (C&D) The 5637 cells were treated with indicated dose of THE NPs for 24 h, and were subject to ICC analyses.

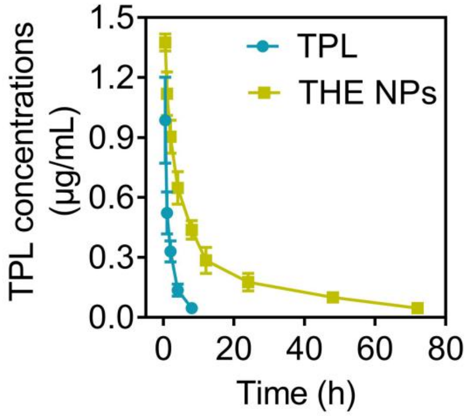

Materials	AUC	T-half (h)
**TPL**	1.638	0.9
**THE NPs**	14.69	4.9

Figure S19. The improved bioavailability by in vivo pharmacokinetics: 6 mice (FVB, female 6-week old) were randomly assigned to 3 groups (n = 3). 1.05 mg/kg of THE NPs, 0.35 mg/kg of TPL were i.v. administrated to the mice in these groups, respectively. The blood was collected at different times from the tail vein. 100 μL blood was collected each time and extracted with chloroform (3 x 1 mL), followed by evaporation of chloroform, and then dissolved with 1 mL of chromatographically pure methanol, filtered and detected by HPLC. HPLC conditions: acetonitrile/water (15:85) solution as mobile phase, 1 mL/min, retention time 36.8 min, UV210 nm.

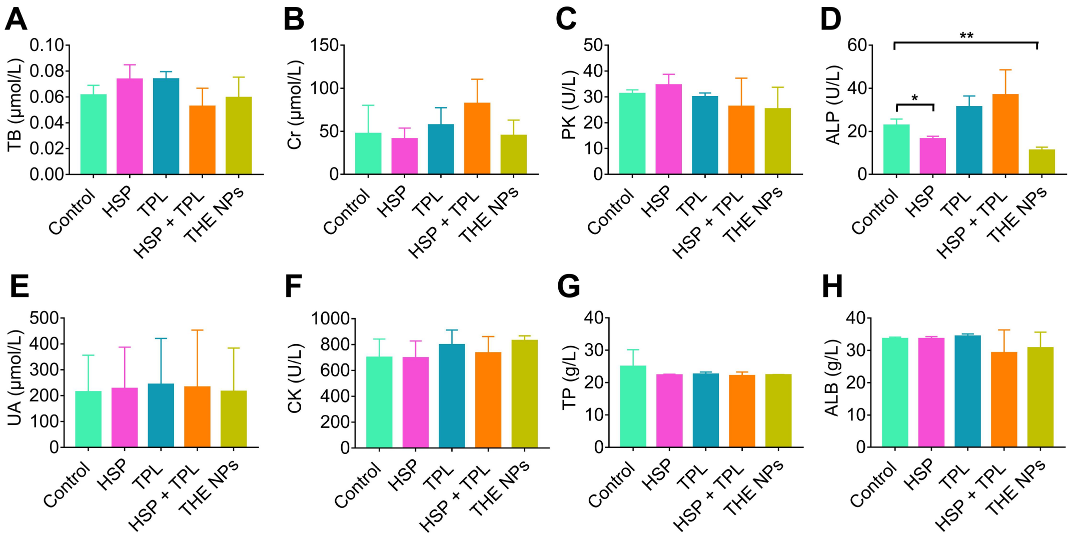

Figure S20. Test results of biochemical indexes of C57 mice

(A-H) The TB, Cr, PK, ALP, UA, CK, TP and ALB level in C57 mice after treating with PBS, HSP, TPL, HSP + TPL or THE NPs for 24 h.
